# High‐Throughput Tiling of Essential mRNAs Increases Potency of Antisense Antibiotics

**DOI:** 10.1002/advs.202504284

**Published:** 2025-04-30

**Authors:** Giorgia Danti, Linda Popella, Jörg Vogel, Hans M. Maric

**Affiliations:** ^1^ Rudolf Virchow Center for Integrative and Translational Bioimaging University of Würzburg 97080 Würzburg Germany; ^2^ Institute for Molecular Infection Biology (IMIB) Faculty of Medicine University of Würzburg 97080 Würzburg Germany; ^3^ Cluster for Nucleic Acid Therapeutics Munich (CNATM) 80802 Munich Germany; ^4^ Helmholtz Institute for RNA‐based Infection Research (HIRI) Helmholtz Centre for Infection Research (HZI) 97080 Würzburg Germany

**Keywords:** acpP, Antisense Antibiotics, Asobiotics, ftsZ, mRNA, murA, PNA, rpsH

## Abstract

Antimicrobial resistance is outpacing drug discovery, creating an urgent need for precision‐based strategies to counteract resistant pathogens. Peptide nucleic acid (PNA)‐based antisense molecules offer a promising approach by selectively inhibiting essential bacterial mRNAs, but their design rules for optimal efficacy remain incompletely understood. Here, a scalable high‐throughput platform is developed for the nanomolar‐scale one‐shot synthesis of PNAs as carrier peptide conjugates (PPNAs). Parallel synthesis of up to 1,536 PPNAs composed of up to 21 PNA or peptide building blocks enabled systematic, base‐by‐base analysis of RNA hybridization, mRNA inhibition, and antimicrobial activity across nine essential genes in uropathogenic *Escherichia coli*. The accuracy and robustness of this high‐throughput tiling platform are demonstrated through in‐depth analysis of the *acpP* mRNA and identify potent antisense inhibitors of *rpsH*, *ftsZ*, and *murA*. This approach provides an efficient and scalable route to design and optimize PNA‐based antimicrobials, facilitating empirical testing across diverse bacterial targets. By enabling large‐scale exploration of the relevant mRNA sequence space, the sequence tiling platform accelerates the discovery of antisense‐based antimicrobials, offering a scalable strategy to develop precision therapies against various pathogens and combat resistance.

## Graphical abstract

The systematic tiling of essential genes’ mRNA here presented, proposes a valuable tool for the identification of novel PNA sequences with antibiotic potential. The high‐throughput synthetic set up opens the door to investigating thousands of sequences in an economic way and ultimately identifies potent antisense oligonucleotides while also giving room to explore potentially interesting modifications.

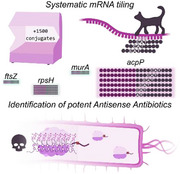

## 1 Introduction

The rising threat of antibiotic‐resistant bacteria and their potential to jeopardize the success of modern medicine underscore the critical need for innovative antimicrobial solutions. Among these, programmable antisense oligomers (ASOs) stand out as a promising class of antibiotics, capable of targeting bacterial mRNAs in a sequence‐specific and thus potentially species‐specific manner.^[^ ^1^, ^2^, ^3^, ^4^, ^5^ ^]^ Such ASO‐based antibiotics exert their action through inhibiting mRNA translation, thus suppressing the synthesis of encoded essential proteins. Their activity has been proven against several bacteria, with oligomers of peptide nucleic acid (PNA) and phosphorodiamidate morpholino (PMO) being the most popular modalities. In PNA, the phosphate ribose backbone is replaced with a modified N‐(2‐aminoethyl)glycine peptide backbone, and nucleobases are attached without the sugar component. PNA oligomers are non‐toxic and non‐immunogenic and resistant to degradation by nucleases or proteases.^[^ ^3^, ^4^, ^6^ ^]^ They are broadly used as probes and diagnostics^[^ ^7^, ^8^, ^9^, ^10^, ^11^ ^]^ and increasingly studied as antimicrobial agents.^[^ ^12^, ^13^ ^]^ While they have not progressed to the stage of drug approval, they have shown remarkable growth inhibitory activity against Gram‐negative and, to a lesser extent, Gram‐positive bacteria.^[^ ^2^, ^3^, ^14^, ^15^ ^]^ The neutral backbone of PNA facilitates effective target hybridization at minimal size due to the lack of ionic charge repulsion.^[^ ^3^, ^16^, ^17^ ^]^ Delivery has been achieved by different carriers, with cell penetrating peptides (CPP) composed of cationic and hydrophobic amino acids being most common. The efficacy of the CPP‐mediated uptake varies among bacterial strains and different CPPs.^[^ ^18^, ^19^, ^20^, ^21^ ^]^

To improve the efficacy of antisense antibiotics, several aspects must be addressed. Not only how the ASO reaches and enters the bacterium of interest, but also the sequence requirements for effective target mRNA engagement. At present, few if any design rules that balance the four underlying aspects of delivery to the site of action, cellular uptake, target hybridization, and ensuing toxic off‐target effects exist, but can likely be inferred from empirical screens that test PNA efficacy at large scale. However, such screens have suffered from the prohibitive cost of producing hundreds if not thousands of PNA variants. In addressing this, we present a new experimental approach for high‐throughput synthesis and direct evaluation of PNAs to facilitate the optimization of antibacterial ASOs for a set of essential mRNA targets in a bacterial pathogen of concern.

**Figure 1:**
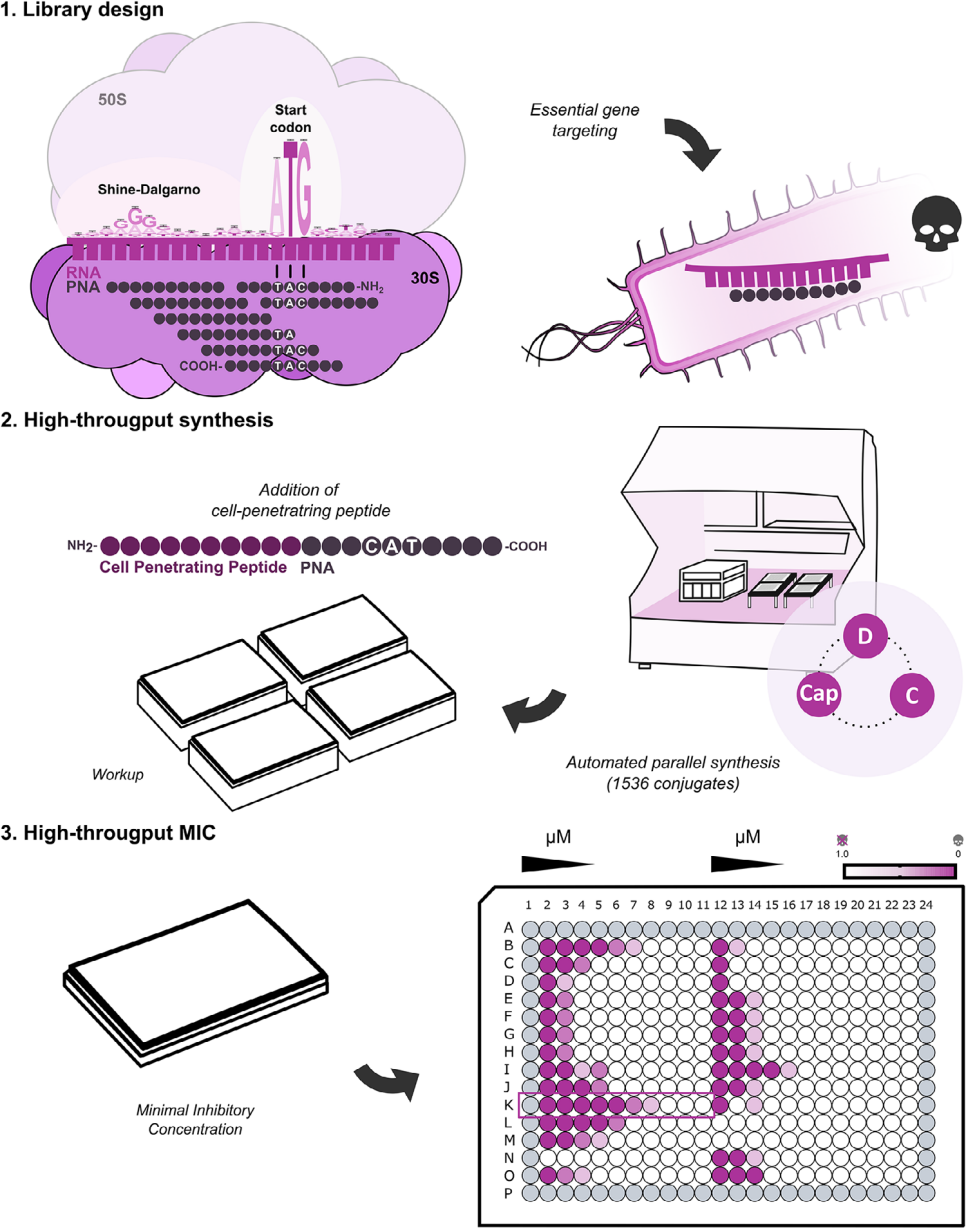
Nanomolar‐scale direct‐to‐biology screening approach. Schematic representation of the general workflow. 1). Starting from the selected (essential) target mRNA (pink), a library of PNA binders is created with a 1 base‐pair (bp) offset along the TIR, including the translational start codon “AUG”, which translates into “CAT” in the respective PNA antisense sequences. 2). PNAs were produced in parallel, in an automated cellulose‐based set up. 3). For testing the efficacy of bacterial growth inhibition, PNAs are directly synthesized as CPP‐conjugates, i.e., fused to (KFF)_3_K, and subjected to broth microdilution assays for determination of their minimal inhibitory concentrations.

The primary mode of action of antibacterial ASOs is the steric exclusion of 30S ribosomes, which recognize the translation initiation region (TIR) of bacterial target mRNAs^[^ ^22^ ^]^ to initiate protein synthesis. To this end, ASOs are typically designed to base pair within the TIR spanning the mRNA's start codon (most often AUG) and the Shine‐Dalgarno sequence (**Figure** **1**). Functional and structural data indicate that the physical contact area between the 30S subunit, initiator tRNA, initiation factors and the mRNA spans ≈50 nucleotides (nt) in *E. coli*.^[^ ^23^, ^24^, ^25^, ^26^, ^27^ ^]^ The interaction window is further confirmed by work on bacterial regulatory small RNAs, which typically must hybridize within 15 nt upstream or downstream of the start codon to inhibit translation.^[^ ^28^, ^29^ ^]^ Consequently, this sequence space lends itself for a proof‐of‐concept study applying high‐density ASO tiling of the TIR and early coding sequence of target mRNAs. It also lends itself to systematic testing of the influence of sequence length and composition^[^ ^6^, ^15^ ^]^ on ASO uptake and the efficacy of target mRNA inhibition.

While there is a clear need for systematic characterization of ASO properties, such efforts have been hampered by the lack of cost‐effective methods for a in high‐throughput synthesis of large numbers of PNA or PMO‐based ASOs, of sufficient amount for antibacterial efficacy determination. Despite the introduction of PNA more than 30 years ago,^[^ ^7^ ^]^ PNA preparation remains more challenging^[^ ^30^ ^]^ than synthesizing conventional peptides. PNA monomers and their polymers exhibit poor water solubility, resulting in less efficient coupling and a tendency to self‐aggregate on resin, commonly restricting PNA to short sequences and limiting high‐throughput synthesis. Alternative protection groups have overcome some of these limitations.^[^ ^7^, ^31^, ^32^, ^33^, ^34^, ^35^, ^36^, ^37^, ^38^, ^39^, ^40^, ^41^, ^42^, ^43^, ^44^, ^45^, ^46^, ^47^, ^48^, ^49^, ^50^ ^]^ In addition, the CPP and PNA moieties might be synthesized separately, followed by a conjugation step.^[^ ^51^ ^]^ This strategy, however, requires additional purification steps during synthesis, thus being incompatible with economic high‐throughput production or direct‐to‐biology screening, i.e., the direct use of a library for activity screening without purification. That being said, the peptide nature of both moieties enables a direct one‐shot synthesis based on Fmoc‐based Solid‐Phase Peptide Synthesis.^[^ ^52^ ^]^ Arguably, the lack of scaled building block production and the high cost of the Fmoc/Bhoc monomers, together with their unfavorable solubility, especially of the C monomer, may have limited broader use of this strategy. In this work, we have overcome these limitations by adapting coupling and scale of synthesis, and turned it into an economic high‐throughput synthesis approach in which the production of bioactive CPP‐PNA (PPNA) conjugates is successfully combined with testing bacterial growth inhibition (Figure 1).

The parallel PPNA synthesis has substantially shortened production time and enabled a systematic screening of PNA efficacies along the TIR of the gold‐standard PNA target *acpP* and eight additional essential mRNAs in uropathogenic *E. coli* strain 536 (UPEC 536). Base‐by‐base analysis and length variations yielded a total of 290 PPNAs that were subjected to bacterial growth inhibition assays and 366 PNA constructs that were tested in hybridization arrays in vitro. The robustness and accuracy of this PPNA production platform, to which we will refer as sequence tiling, was confirmed by side‐by‐side comparison with commercially produced purified PPNAs and aligns with previously reported growth inhibiting PNA sequences. Combined with predictions by the MASON software^[^ ^53^ ^]^ for PNA design, this experimental platform will accelerate the discovery of novel targetable bacterial mRNAs and the identification of optimal antisense PNA sequences.

## 2 Results and Discussion

PNA libraries have been produced and studied in various formats, using purification‐free,^[^ ^54^ ^]^ selective immobilization^[^ ^55^ ^]^ or light‐directed synthesis approaches.^[^ ^56^ ^]^ However, they have not been used for comprehensive PNA/RNA hybridization studies or functional read‐outs such as growth inhibition of CPPPPNAs in a high throughput approach.

### 2.1 High‐Throughput Synthesis of PNAs in µSPOT Format

**Figure 2:**
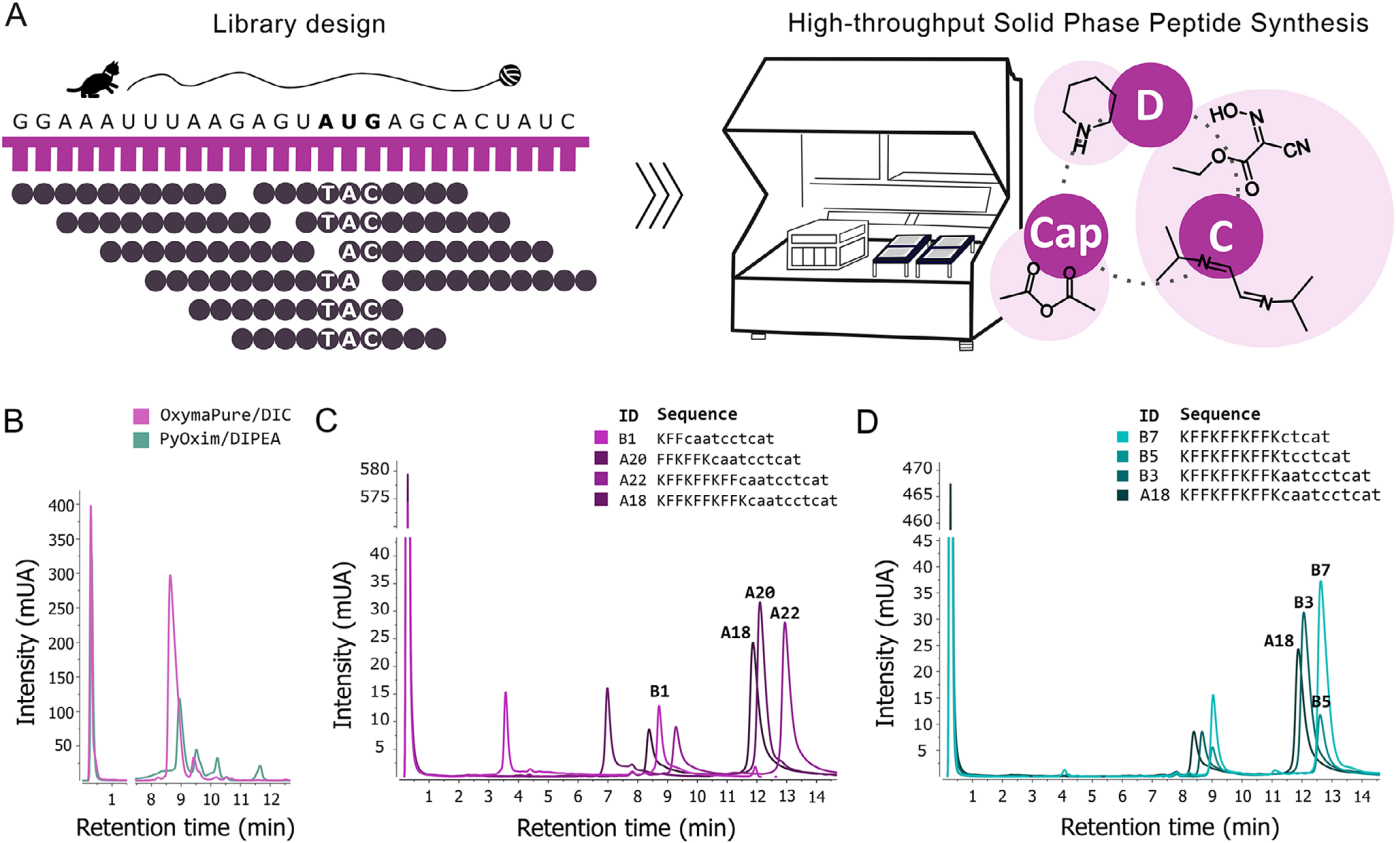
Coupling conditions for automated parallel CPP‐PNA (PPNA) conjugate synthesis. A). Schematic representation of PNA design and synthesis. PNA sequences are designed to hybridize along the target mRNA's TIR (here exemplarily shown for *acpP*) encompassing the translational start codon “AUG” which translates into “CAT” in the cognate PNA sequence. PNAs are then synthesized on a highly‐derivatized cellulose disc (*n* = 100 nmol) support following the outlined coupling, capping, and deprotection cycle. PPNAs are synthesized from C to N termini and cleaved in parallel in 96‐well format. B). Comparison of coupling chemistry in parallel PNA synthesis. In the prolonged parallel PNA synthesis, purities and yields are superior with the more stable and less reactive OxymaPure/DIC compared to PyOxima/DIPEA. C,D). Systematic analysis of the effect of truncation on yields. ©. Truncation of Cell Penetrating Peptide (CPP). (D). Truncation of Peptide Nucleic Acid (PNA). Sequence length of the conjugate does not negatively affect synthesis yield. Major side products are the capped/acetylated truncations.

Here, we adapted and advanced previous protocols^[^ ^54^, ^55^, ^57^ ^]^ using Ronald Frank's SPOT approach^[^ ^58^ ^]^ for high‐nanomolar scale one‐shot synthesis of PPNAs using commercially available Fmoc/Bhoc monomers at low (0.1 m) concentration, achieving parallel synthesis of up to 1536 unique sequences (**Figure** **2**A). Previous studies reported highly reactive coupling reagents to be optimal for complete PNA‐building block coupling at room temperature (rt) with short coupling times.^[^ ^59^ ^]^ Specifically, PyOxim/N,N‐Diisopropylethylamine (DIPEA) gave best yields due to the reduced N‐terminal tetramethylguanidinium byproduct formation compared to the traditionally used phosphonium salts such as HOAt^[^ ^54^ ^]^ or HATU. However, a direct comparison of coupling agents, identified the less reactive OxymaPure/N,N‘‐diisopropylcarbodiimide (DIC) as the most optimal reagent for high‐throughput nanomolar‐scaled parallel synthesis (Figure 2B). Here, the prolonged stability of the less reactive OxymaPure/DIC reduced hydrolysis and oxidation, thus leading to superior performance while avoiding major side‐reactions. Notably, this protocol achieved high coupling efficiencies (>97%) even on highly derivatized cellulose matrix,^[^ ^57^ ^]^ qualifying it for array‐based synthesis and subsequent testing in broth microdilution assays.

Next, we explored the limits of our synthetic set up by performing systematic truncation of an exemplary test sequence,^[^ ^14^, ^15^, ^60^ ^]^ namely three‐ to ten‐mer ((KFF)_3_K) peptide variants followed by five‐ to ten‐mer PNA sequences (“caatcctcat”) (Figure 2C,D). Of note, previous work had identified (KFF)_3_K as optimal delivery CPP for PNA delivery into *Salmonella* and UPEC 536.^[^ ^14^, ^15^, ^61^, ^62^, ^63^ ^]^ Sequences were effectively cleaved from the solid support via a C‐terminal Rink‐amide building block and analyzed via LC‐MS. Importantly, increasingly incomplete coupling steps or major side‐product formation due to aggregation^[^ ^30^ ^]^ would result in rapidly dropping yields with increasing sequence length. Yet, HPLC analysis confirms that yields are independent from the here studied PNA and peptide lengths using the outlined chemistry and coupling protocol (Figure 2C,D and Method Section). Taken together, this establishes technical feasibility of the parallel synthesis of up to 1536 PNA‐peptide conjugates at 100 nmol scale within the range of the tested sequence lengths.

### 2.2 Systematic Biological Screening of *acpP*‐Targeting PNAs

**Figure 3:**
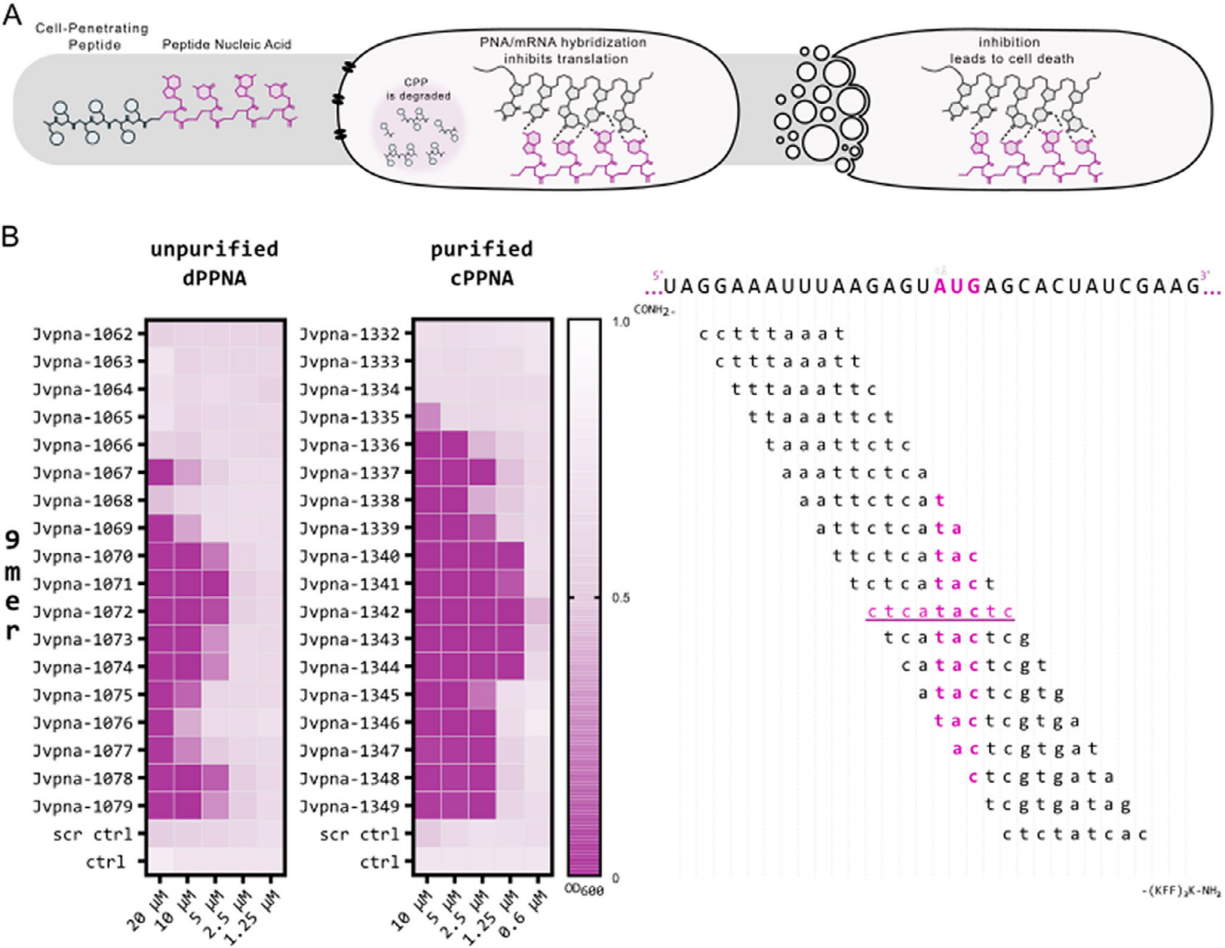
MIC assays of our disc‐based PPNA (dPPNA) versus commercial PPNA (cPPNA) constructs. A). Schematic representation of the cellular mode of action of PPNAs in bacteria. The CPP mediates cell entry and dependent on the CPP used, here (KFF)_3_K, it undergoes degradation in the extracellular or periplasmic space. In the cytoplasm, the PNA hybridizes with its target mRNA and based on the binding region leads to the inhibition of translation. When targeting essential bacterial mRNAs this ultimately causes bacterial death. B). Direct‐to‐biology validation against UPEC 536 used at 10^5 cfu mL^−1^ in Mueller Hinton broth. Determination of the PPNAs MICs in a 384 well plate setup. Comparison of disc‐produced and HPLC‐purified conjugates titrated from 20–1.25  to 10–0.6 µm, respectively. A scrambled PNA control (“cactatctc”) was included. The lowest concentration of a PNA that inhibits growth, i.e., with a final OD_600_ of ‘<0.05′, indicates the MIC. All PNA sequences are shown and their conjugation to the cell penetrating peptide (KFF)_3_K is indicated. The constructs JVpna‐1763 and JVpna‐178 are scrambled sequence controls (scr ctrl) for each of the two PPNA sets. Water was added in an equal volume serving as growth control (ctrl). The final OD at 600 nm after 24 h is shown in a white‐to‐magenta color gradient. Grey cells indicate conditions that were not tested. MIC testing for dPPNA constructs was performed two to five times, and two times for all cPPNA constructs.

As validation, we tested the antibacterial activity of our disc‐derived CPP‐conjugated PNAs (dPPNAs) compared to commercially available counterparts (cPPNAs, **Figure** **3**; Peps4LS GmbH). Apart from the different synthesis platforms, i.e., paper‐disc versus peptide synthesizer, the cPPNAs but not the dPPNAs were subjected to HPLC purification. Thus, dPPNAs have a generally lower purity, with expected weaker antimicrobial activity as judged by higher minimal inhibitory concentrations (MICs). To determine the MIC of each PPNA, we performed susceptibility screenings with UPEC 536 using 384 microtiter plates. We selected the essential *acpP* gene as the target (Figure 3A) and synthesized all possible complementary 9mer PNA antisense sequences to tile the TIR of the *acpP* mRNA in one‐base steps from positions ‐14 to +12 relative to nucleobase “A” of the “AUG” translational start codon (Figure 3B). For efficient bacterial uptake, the PNAs were directly fused to the CPP (KFF)_3_K during synthesis.

MIC assays with the above library gave two main results (Figure 3B). First, we recapitulated the published antibacterial activities^[^ ^15^ ^]^ against UPEC 536, while observing overall higher MICs (ca. 4‐ to 8‐fold) of our dPPNAs relative to the respective cPPNAs. Second, relative growth inhibitory capacities in both sequence tiling experiments were replicating the same sequence‐to‐activity relationship for the dPPNAs and cPPNAs. We therefore conclude that the disc‐based PPNA synthesis approach offers a cost‐effective scalable alternative for screening a large set of different genes and PPNAs.

### 2.3 Finding the Sweet Spot – PNA Target Window versus PNA Length Restriction

**Figure 4:**
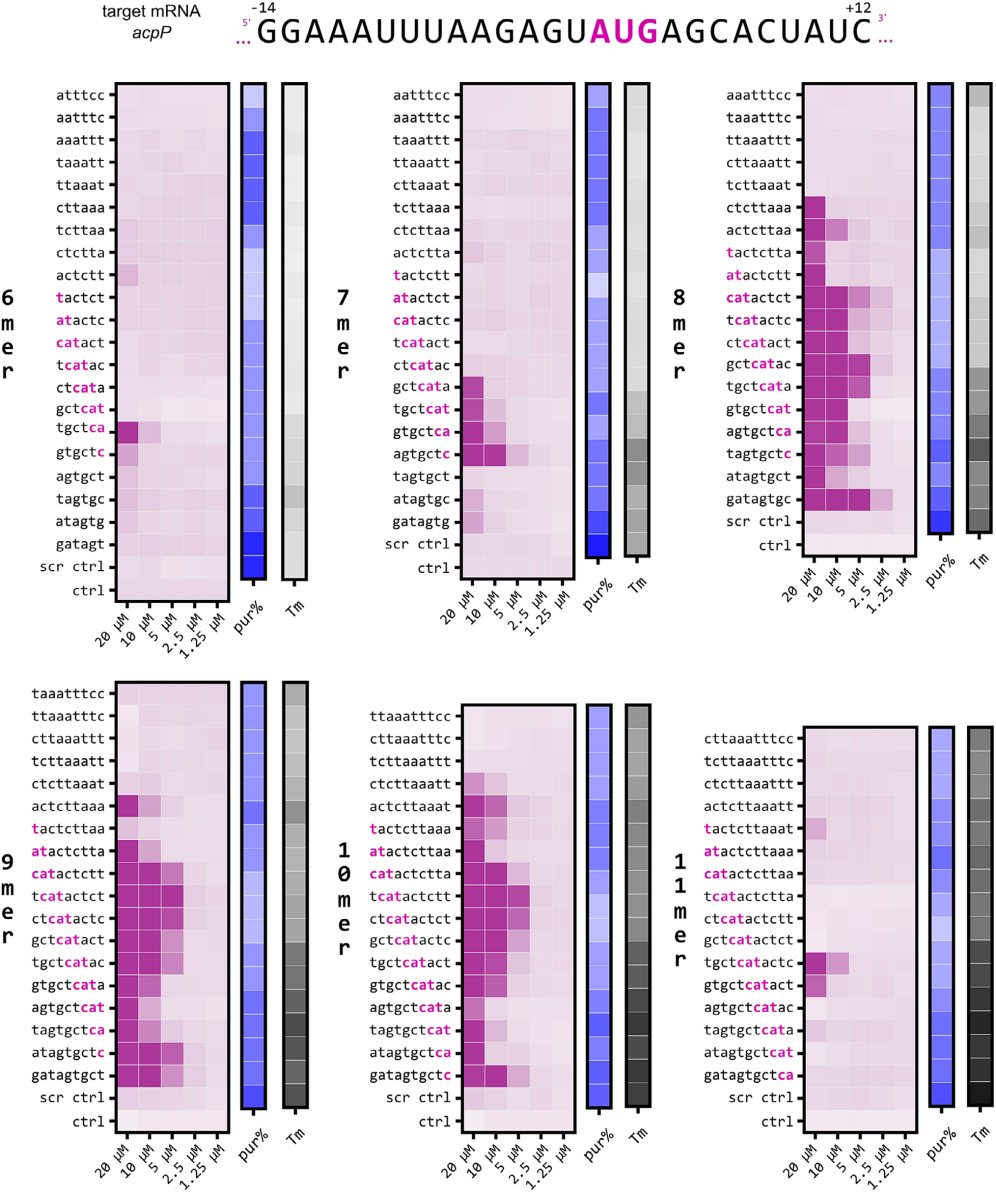
Disc‐based PNA synthesis platform enables simple identification of optimal PNA sequences with minimal length requirements (*acpP*). Direct‐to‐biology validation of 6–11mer PNA sequences complementary to the *acpP* target mRNA against UPEC 536 used at 10^5 cfu mL^−1^ in Mueller Hinton broth. Magenta‐white heatmaps show the final optical density (OD_600_) at 24 h post treatment together with each PNA's purine content (blue‐scale) and its predicted melting temperature (grey‐scale; https://www.pnabio.com/support/PNA_Tool.htm). The lowest concentration of a PPNA that inhibits growth, i.e., with a final OD_600_ of “<0.05′, indicates the MIC. Scrambled PPNA controls (2nd last row per panel) were included for each set of PPNAs. MIC assays were performed at least two times and the average OD_600_ is shown.

Apart from the actual antisense PNA sequence, the length of the PNA is an additional critical factor for its antimicrobial activity,^[^ ^6^, ^15^ ^]^ because it influences uptake, binding strength, and off‐targeting (selectivity). Specifically, 9 and 10mer PNAs generally show optimal growth inhibition, while PNAs of ≤8 or ≥11 nucleobases usually show lower antibacterial activity.^[^ ^14^, ^15^, ^63^ ^]^ This can be rationalized by weaker binding strength of shorter PNAs and a poorer uptake of longer PNAs. Due to the allowance of flanking mismatches, PNAs with ≥10 nucleobases may have broader off‐targeting activity than their shorter counterparts with ≤9 nucleobases.^[^ ^53^ ^]^ Here, we use our disc‐based synthesis platform and sequence tiling to generate and systematically screen antimicrobial activity of 6 to 11mer *acpP*‐targeting PPNAs (**Figure** **4**). We observed that 8–10mer PNAs show optimal antibacterial activities. A similar trend was observed with a second target, the *rpsH* mRNA (ribosomal protein S8), albeit with a narrower efficacy window (Figure S5, Supporting Information). Thus, this screen recapitulates expected activity profiles of two previously reported PNA targets, and differences in the activity profiles for 8mer and 11mer *acpP* PNAs.^[^ ^6^, ^15^, ^63^ ^]^ Accordingly, our sequence tiling approach lends itself for pre‐screening of PNA sequences for optimal targeting.

### 2.4 Determination of PNA‐Based Blockers for Eight Additional Essential Bacterial Genes

To validate our disc‐based PNA synthesis platform with more targets, we chose seven additional essential genes with diverse functions. Some of these are known targets of conventional antibiotics, others are established to be susceptible PNA targets. These were tested with 9mer PNA sequences, again used as (KFF)_3_K‐conjugates. Specifically, we targeted the following cellular functions: cell division (*ftsZ*; encoding a protein that forms a ring at the site of cell division and a known potent PNA target in UPEC 536^[^ ^15^ ^]^); DNA replication and repair (*gyrA*, encoding a DNA gyrase subunit essential for supercoiling and replication of DNA and a known PNA target in several different bacteria;^[^ ^64^ ^]^ and *parE*, encoding a subunit of topoisomerase IV, crucial for chromosome segregation and untangling DNA) both pathways targeted by fluoroquinolones; transcription (*rpoB* encoding the beta subunit of RNA polymerase, essential for DNA transcription), a process targeted by Rifampicin or Fidaxomicin, and metabolic genes (*folA*, encoding dihydrofolate reductase, essential for folate metabolism and nucleotide biosynthesis which is conventionally disrupted by Trimethoprim; *lpxC*, involved in the first step of lipid A biosynthesis, crucial for cell membrane integrity and blocked by pre‐clinical LpxC inhibitors; and *murA*, which encodes an enzyme involved in peptidoglycan synthesis, essential for cell wall construction). *murA* is a known drug target of Fosfomycin and a promising PNA target in *E. coli, S. enterica*, and *K. pneumoniae*.^[^ ^65^, ^66^ ^]^

**Figure 5:**
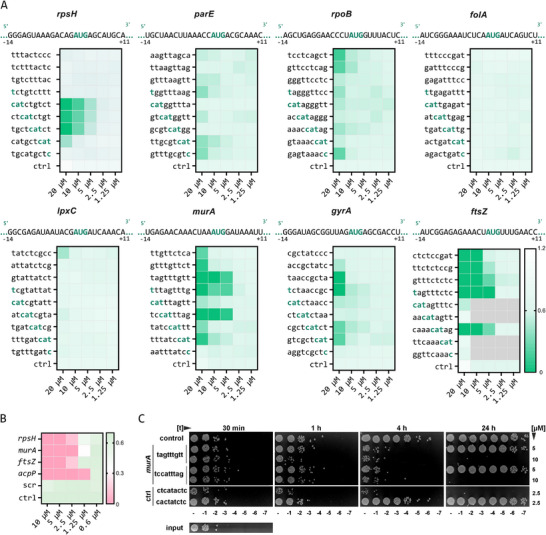
Sequence tiling of additional essential bacterial genes known to be druggable by conventional antibiotics or PPNAs. A). Systematic PNA sequence tiling analysis of drug‐related target genes or known PNA targets using dPPNAs. Sequences were designed to be complementary to the translation initiation region of the respective target mRNA within a window spanning nucleotides −14 to +11 each with a 2 bps offset. PPNAs were tested against UPEC 536 (10^5 cfu mL^−1^) in Mueller Hinton broth and the final OD_600_ after 24 h is shown as green‐white heatmap. MIC assays were performed at least two times and the average OD_600_ is shown. B). Validation of the growth inhibitory activity of selected dPPNA hits targeting *rpsH, murA*, and *ftsZ*. Respective PPNAs were purchased in HPLC‐grade from Peps4LS GmbH (cPPNA). The gold standard *acpP* cPPNA was included as positive control, while a sequence unrelated cPPNA was added as scrambled control (scr). Water was added in an equal volume serving as growth control (ctrl). cPPNAs were titrated from 10 to 0.6 µm and tested against UPEC 536 (10^5 cfu mL^−1^) in Mueller Hinton broth. The final OD at 600 nm after 24 h is shown in a pink‐to‐green color gradient. MIC testing was performed four times and the average OD is shown. The lowest concentration of a PPNA that inhibits growth, i.e., with a final OD600 of “<0.05”, indicates the MIC (summarized in Table S3, Supporting Information). C. UPEC 536 was treated with the indicated *murA*‐targeting dPPNAs at 5  and 10 µm for the time periods shown above. An *acpP*‐targeting cPPNA (‘ctcatactc”, JVpna‐177, 2.5 µm) and a respective scrambled cPPNA (“cactatctc”, JVpna‐178, 2.5 µm) were used as an internal positive and negative control, respectively. One representative example out of two spotting assays is shown.

The outcome of this extended sequence tiling screen is summarized in **Figure** **5**. For three genes (*rpsH, ftsZ* and *murA*) we identified PNA sequences with outstanding growth inhibitory potency, comparable to that of *acpP*‐targeting PNAs (Figures 4 and 5; Figure S5, Supporting Information). Particularly, *rpsH*‐targeting JVpna‐1105 “*catctgtct*”’, *murA*‐targeting JVpna‐1135 “tagtttgtt” and JVpna‐1138 “tccatttag”, and *ftsZ*‐targeting JVpna‐1145 “tagtttctc” and JVpna‐1148 “caaacatag” were identified as potent growth inhibitors and the individual optimal binding hotspots have been narrowed down for these targets.^[^ ^14^, ^65^, ^66^ ^]^ The growth inhibitory capacity of selected sequences was further validated with their respective HPLC‐purified counterparts (Figure 5B). A bactericidal activity of the two top PNA sequences directed against *murA* (JVpna‐1135 “tagtttgtt”, JVpna‐1138 “tccatttag”) was additionally confirmed in spotting assays (Figure 5C). We observed delayed killing of the *murA*‐targeting PNAs compared to the *acpP*‐targeting counterpart, but with long‐lasting bactericidal activity until 24 h. By targeting *gyrA, parE* and *rpoB* we identified PNA sequences that inhibit growth with intermediate MICs. By contrast, targeting of *folA* and *lpxC*, two previously untested PNA target mRNAs, did not result in detectable growth inhibition within the studied range of concentrations. This might be due to an insufficient knockdown of the respective mRNAs. Nonetheless, the data presented here prove the value of our nanomolar‐scale direct‐to‐biology screening approach for the efficient identification of susceptible PNA target mRNAs and to narrow down the optimal ASO binding site for superior bacterial growth inhibition.

### 2.5 RNA/PNA Hybridization in µSPOT Microarray Format

**Figure 6:**
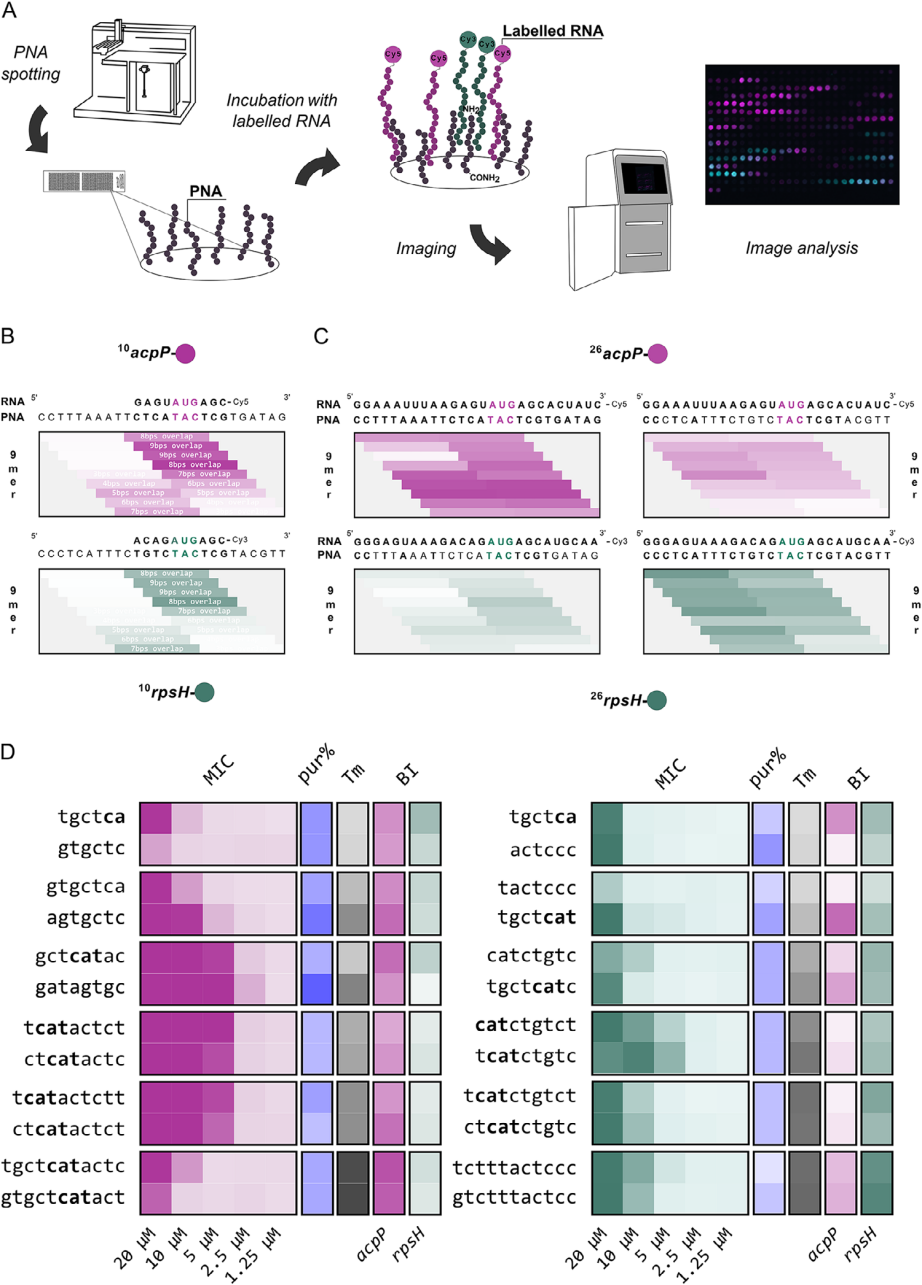
Microarray PNA/RNA hybridization study. A). Schematic representation of the PNA/RNA hybridization read‐out. Microarray slides with immobilized *acpP* and *rpsH* PNA sequences are incubated with fluorescently 3′‐labeled template RNA probes and then imaged for binding strength quantification. Simultaneous incubation with differently labeled *acpP‐* or *rpsH‐*specific RNA probes mirrors binding competition and specificity. B). Exemplary *acpP*‐specific hybridization outcome for a 10 nt *acpP* Cy5 3′‐labeled RNA probe (top), in presence of the respective Cy3 3′‐labeled *rpsH* probe (bottom) with noted bases overlap between probe and PNA sequence displayed on the array. C). *acpP* (top) and *rpsH* (bottom)‐specific hybridization outcome for 26 nt 3′labeled‐RNA probes. On the left, *acpP*‐based PNA sequence are displayed, meanwhile *rpsH*‐based PNA sequences are displayed on the right. Each microarray displays the library in duplicate. The experiments were performed three times independently and the average binding intensities are shown. C. Summary of MIC, purine content, melting temperature, and normalized binding intensities (BI) results for best performing sequences for each PNA length, based on MIC outcomes.

The sequence tiling of nine mRNAs in total revealed a complex sequence activity profile. To clarify a possible contribution of the underlying PNA/RNA hybridization strength or resolve possible synergistic or depleting RNA binding events we next studied PNA/RNA binding in microarray format. The presence of secondary structure elements within the PNA binding region of the target RNA, the difference in the backbone architecture of RNA versus PNA, and tolerated mismatches in a PNA/RNA duplex can be expected to result in complex hybridization dynamics. While PNA microarrays have previously been used to study the effect of mismatches,^[^ ^67^ ^]^ large‐scale PNA/RNA hybridization studies that might identify optimal PNA sequences and inform advanced melting temperature prediction algorithms are still lacking. To establish an accessible, economic, and systematic assessment of RNA/PNA hybridization in high‐throughput, we produced PNA microarray copies using the µSPOT method.^[^ ^57^ ^]^ Notably, PNAs are handled and printed as cellulose conjugates from DMSO stocks, thereby eliminating interference by aggregation or precipitation. PNA microarrays were incubated with fluorescently labeled RNA probes to visualize hybridization strength and specificity/selectivity (**Figure** **6**A).

We investigated the binding of various *acpP* mRNA‐derived probes (10, 26, 18, and 80 nt, Figures S1–S3, Supporting Information) to identical replicates of microarrays displaying all possible complementary 6‐, 7‐, 8‐, 9‐,10‐ and 11‐mer PNA sequences, summing up to ∼1800 PNA/RNA hybridization events in total. To control for potential interference of the fluorescent dye with the immobilization surface, e.g., through steric hindrance or electrostatic interaction, we compared 3′‐labeled (Figure S1A, Supporting Information) and 5′‐labeled RNA probes (Figure S4, Supporting Information). Hybridization of the 5′‐labeled RNA with the immobilized PNA sequences would bring the dye in proximity to the microarray surface, whereas the 3′‐attached fluorescent label would be solvent‐exposed. While we did not observe dye‐position dependent effects, the hybridization window correlated with the length of the RNA probe. We observed bell‐shaped curve interaction windows that were centered to the PNA sequences possessing complete complementarity. For longer RNA probes, a bias toward the 5′ of the template sequence for hybridization strength was observed (Figure S3A, Supporting Information), possibly resulting from increasing steric hindrance between the unbound overhanging RNA and the array surface in this orientation.

### 2.6 Array‐Based Study of RNA/PNA Target versus Off‐Target Binding

Hybridization studies in our microarray format could resolve possible target versus off‐target gene hybridization and resolve competition events. Thus, we employed our microarray‐based screening to systematically analyze off‐target hybridization of PNAs complementary to *rpsH* with *acpP*‐derived RNA probes and vice versa (Figure 6A). To this end, we used the above PNA libraries for tiling the TIRs of *acpP* and *rpsH*. These libraries were then simultaneously incubated with 10, 26, or 80‐nt, *acpP* and *rpsH* RNA probes that were 3′‐labeled with Cy5 or Cy3, respectively (Figure 6B,C; Figures S1 and S3, Supporting Information). We also noticed that the target sequence of the *rpsH* TIR shows 100% and 64% similarity to the corresponding 7mer and 11mer PNA for *acpP*, respectively. Simultaneous incubation with Cy5‐*acpP* RNA and Cy3‐*rpsH* RNAs resolved target over off‐target binding. Under the tested conditions, three and four mismatches in the 10mer and 11mer sequences sufficed to ensure target‐gene hybridization specificity. In contrast, the 9mer (78% similar), 8mer (88% similar) and 7mer (100% similar) PNAs showed increasing cross‐reactivity, suggesting a potentially cross reactivity on both genes in vitro. Ultimately, this approach provides valuable hybridization information that can be easily put in correlation to many parameters, such as purine content, predicted melting temperature, and MIC values (Figure 6D).

## 3 Conclusion

To properly address and identify requirements for potent essential mRNA targeting with PNAs, access to large‐scale PNA libraries is essential. Recently, we highlighted the relationship between PNA efficacy in inhibiting translation in vitro and their predicted hybridization temperature (T_m_s), as well as the importance of considering transcriptome‐wide off‐targeting in PNA design.^[^ ^53^ ^]^ However, these parameters alone cannot accurately predict the most effective PNA sequences for a given target mRNA, making experimental validation essential. The high‐throughput platform for PNA synthesis and direct‐to‐biology testing developed here, for the first time, enabled a comprehensive analysis of RNA hybridization in vitro and growth inhibition in vivo. Despite identifying specific sequences with efficient essential mRNA binding and potent antibacterial activity, our high‐throughput tiling approach highlights the complexity of PNA binding hotspots across different target mRNA TIRs, revealing no simple position‐dependent overlap. Notably, for both *ftsZ* and *murA*, so far used PNAs consistently targeted the same suboptimal frame directly on the start codon.^[^ ^15^, ^62^, ^65^ ^]^ Crucially, for *ftsZ* and *murA*, our tiling approach reveals that shifting the targeting region beyond the AUG significantly improves potency. Thus, high‐throughput screens are needed to better understand PNA activity profiles and to guide PNA design. Generally, PNA efficacy is a complex interplay of different parameters including uptake efficacy, target hybridization strength or the off‐target range, which could lower the availability of the PNA for its actual target. Additionally, self‐complementarity of the PNA itself plays a critical role in PNA efficacy and should be avoided. Our high‐throughput tiling platform will help to improve PNA design with the long‐term goal of achieving precision killing or tailored manipulation of microbes. Synergistic or additive effects of defined PNA cocktails targeting genes within the same or distinct pathways can be easily addressed with a more rapid turnaround time. Additionally, the inclusion of modified PNA building blocks, such as γ‐modified backbones, into the synthesis pipeline can yield PNA constructs with reduced off‐target binding, largely improved target hybridization and widely enhanced growth inhibition. Moreover, PNA‐derivatization cannot only facilitate cell permeability by equipping the PNA with an inherent net positive charge but might also promote new and favorable molecular interactions enhancing blocking capacity and sequence specificity.^[^ ^68^, ^69^ ^]^ Finally, as antimicrobial resistance continues to erode the efficacy of existing antibiotics, innovative approaches that enable rapid discovery of novel therapeutics are urgently needed. By facilitating the empirical identification of effective antisense sequences across diverse bacterial targets, our high‐throughput platform provides a powerful tool to accelerate the development of precision antimicrobials that can circumvent traditional resistance mechanisms. Given that PNA‐based antimicrobials act through sequence‐specific inhibition of bacterial mRNAs, they may offer a viable strategy against multidrug‐resistant pathogens, including those resistant to conventional small‐molecule antibiotics.

Taken together, our high‐throughput tiling approach, combined with one‐shot synthesis of peptide‐PNA conjugates, proves valuable for systematically identifying druggable target mRNAs and determining optimal antisense sequences along their translation initiation regions. Expanding this approach to a larger panel of target genes, extending the PNA target window around the translation initiation region and combined with the introduction of modified PNA bases^[^ ^70^ ^]^ this may foster the identification of novel and more potent programmable antibiotics.

## 4 Experimental Section

### Materials

Commercially available HPLC‐purified peptide‐conjugated peptide nucleic acids (cPPNA) were purchased from Peps4LS GmbH (Heidelberg, Germany). All PNA sequences were synthesized as a direct fusion construct with the cell penetrating peptide (KFF)_3_K. Quality and purity of the constructs were verified by mass spectrometry and HPLC (purity > 98%). cPPNAs (Table S1, Supporting Information) were dissolved in sterile nuclease‐free ultrapure water and heated at 55 °C for 5 min before use. PPNA concentration was determined with a NanoDrop spectrophotometer (A 260 nm) and calculated based on the sequence‐inherent extinction coefficient. For storage aliquots were frozen at – 20 °C, then thawed at room temperature with subsequent heating at 55 °C for 5 min before preparing working solutions. All pipetting steps were performed using low retention pipette tips and low binding Eppendorf tubes (Sarstedt).

Fluorescently labeled RNA probes Cy5‐^80^ *acpP*, Cy3‐^80^ *rpsH* were purchased from (Eurofins). Fluorescently labeled RNA probes ^10^ *acpP*‐Cy5, ^26^ *acpP‐*Cy5, ^10^ *rpsH*‐Cy3 and ^26^ *rpsH*‐Cy3 were purchased from GenScript. All probes are listed in Table S2 (Supporting Information).

### Parallel Solid‐Phase Peptide Synthesis

Peptide‐PNA conjugates were synthesized using a MultiPep RSi robot (CEM GmbH, Kamp‐Lindford, Germany) on in‐house produced, acid‐labile, amino‐functionalized, cellulose membrane discs containing 9‐fluorenylmethyloxycarbonyl‐β‐alanine Fmoc linkers (average loading: 130 nmol/disc – 4 mm diameter).^[^ ^71^ ^]^ Synthesis was initiated by Fmoc deprotection using 20% piperidine (pip) in dimethylformamide (DMF) followed by washing with DMF and ethanol (EtOH). Peptide chain were elongated using a coupling solution consisting of preactivated building blocks (Amino acids (AAs), 0.5 m, PNA monomers (Pns) 0.1 m) with Oxyma (1 m) and DIC (1 m) in DMF (1:1:1, AA/Pn:Oxyma:DIC). Couplings were carried out for 3 × 30 min, followed by capping (4% acetic anhydride in DMF) and washes with DMF and EtOH. Finally, discs were washed with DMF and EtOH. Synthetic Quality Controls (QCs) were transferred into centrifuge tubes and treated separately, while sample discs were transferred to 96 deep‐well blocks.

For microarray production, the synthesis was finalized by deprotection with 20% pip in DMF (2 × 4 µL disc ^−1^for 10 min each), followed by washing with DMF and EtOH.

### Synthetic Quality Control Workup

Fmoc‐protected QCs from synthesis were transferred to centrifuge tubes and treated with a cleavage solution containing 90% trifluoracetic acid (TFA), 2% dichloromethane (DCM), 5% H_2_O and 3% triisopropylsilane (TIPS) (150 µL) for 2 h at rt. Surfactant was transferred to centrifuge tubes containing 700 µL ice‐cold diethyl ether and precipitated overnight at 20 °C. After precipitation, tubes are centrifuged (13 500 rpm, 4 °C) and the surfactant discarded. Precipitated was washed with 500 µL ice‐cold diethyl ether. Samples were centrifugated and surfactant discarded once more to repeat the washing process again. Precipitates were then left to dry and solubilized in 100 µL of 50:50:0.1 Acetonitrile (ACN):HPLC grade Water (H_2_O):Formic Acid (FA). Samples were analyzed in LC‐MS (Agilent) using C18 Monolyth (Phenomenex) with a 0%–100% Solvent B (95% ACN, 5% H_2_O, 0.1% FA) gradient over 10 min. A double wavelength (215 and 280 nm) is used for UV detection. Solvent A is 5% ACN 95% H_2_O, 0.1% FA.

### PPNA Workup

Dried discs are transferred from the synthetic plate to 96 deep‐well plates and treated, while shaking, with Fmoc deprotection solution (2×150 µL, 20% pip in DMF) followed by washes (2×100 µL EtOH, 2×100 µL DCM). After air‐drying, discs are treated with a cleavage solution, consisting of 90% trifluoracetic acid (TFA), 2% dichloromethane (DCM), 5% H_2_O and 3% triisopropylsilane (TIPS) (150 µL well^−1^) for 2 h at rt. Multiple discs of the same conjugate (4‐5) were incubated together with 250‐−300 µL of the same solution. Surfactant was transferred to centrifuge tubes containing 700 µL ice‐cold diethyl ether (single discs) or 1 mL (4‐5 discs) and let precipitate overnight at −20 °C. After precipitation, tubes are centrifuged (13 500 rpm, 4 °C) and the surfactant discarded. Precipitated was washed with 500 µL ice‐cold diethyl ether. Samples were centrifugated and surfactant discarded, to repeat the washing procedure once again. Precipitates are then left to dry in the open air.

### Microarray Production

Dried discs were transferred to 96 deep‐well plates and treated with sidechain deprotection solution, consisting of 90% trifluoracetic acid (TFA), 2% dichloromethane (DCM), 5% H_2_O and 3% triisopropylsilane (TIPS) (150 µL/well) for 1.5 h at rt. The deprotection solution was removed, and the discs were solubilized overnight at rt, while shaking, using a solvation mixture (88.5% TFA, 4% trifluoromethanesulfonic acid (TFMSA), 5% H_2_O, and 2.5% TIPS, 250 µL well^−1^). The peptide‐cellulose conjugates (PCCs) were precipitated with ice‐cold ether (0.7 mL well^−1^) overnight and spun down at 2000 × g for 10 min at 4 °C, followed by two additional washes of the formed pellet with ice‐cold ether. The resulting pellets were dissolved in DMSO (250 µL/well), then PCC solutions were mixed 2:1 with saline‐sodium citrate (SSC) buffer (150 mM NaCl, 15 mm trisodium citrate, pH 7.0) and transferred to a 384‐well plate. To transfer of the PCC solutions to white coated CelluSpot blank slides (76 × 26 mm, Intavis AG), a SlideSpotter (CEM GmbH) was used. After completion of the printing procedure, slides were left to dry overnight.

### Microarray‐Based Hybridization Assay

Slides were incubated with a 1 nm solution of fluorescently labeled RNA probe in Phosphate Saline Buffer Solution (PBS, 137 mm NaCl, 2.7 mm KCl, 10 mm Na_2_HPO_4_, 1.8 mm KH_2_PO_4_, pH 7.4). Incubated slides are first heated to 70 °C for 10 min on ThermoMixerC (Eppendorf) with a plate adaptor and then incubated at 37 °C for 30 min. Slides are immediately imaged using Amersham ImageQuant800 (Cytiva, Danaher) and evaluated with the open source platform MARTin.^[^ ^72^ ^]^

### Minimum Inhibitory Concentration (MIC) Assay

Uropathogenic *E. coli* strain 536 (UPEC 536) was used for PPNA testing (internal strain number JVS‐12054; NCBI GenBank: CP000247.1). Bacteria were streaked on Luria‐Bertani plates and cultured in non‐cation adjusted Mueller‐Hinton Broth (MHB, BD DifcoTM, Thermo Fisher Scientific) with aeration at 37 °C and 220 rpm shaking. Screening for minimum inhibitory concentrations (MICs) of the PPNA constructs was performed according to the broth microdilution method by the Clinical and Laboratory Standards Institute and a recently published protocol with some modifications (40). Specifically, an overnight culture of bacterial cells was diluted 1:100 in fresh MHB and grown to logarithmic phase (OD600 of 0.5). The obtained bacterial culture was diluted to a final cell concentration of ≈105 cfu mL^−1^ by 1:1800 dilution in fresh MHB. Then, 27 µL of the diluted bacterial solution was dispensed into a 384‐well plate (Thermo Fisher Scientific), along with 3 µL of 10x cPPNA or dPPNA working solutions (ranging from 200  to 6.25 µm). A growth control was prepared by adding an equal volume of sterile water instead of PPNA, whereas scrambled PPNAs served as sequence‐unrelated controls. Plates were incubated in a Synergy H1 plate reader (Biotek) with continuous double‐orbital shaking (237 cpm) at 37 °C for 24 h. After 24 h, OD600 was recorded and the MIC was determined as the lowest concentration of PPNA, which inhibited visible growth in the wells (OD600 < 0.1). The final OD_600_ after 24 h is illustrated in heatmaps and represents the average of at least two biological replicates per condition.

### Spot Plating to Determine Time‐Dependent Bactericidal Effects of *murA*‐Targeting dPPNAs

An overnight culture of UPEC 536 was diluted 1:100 in fresh MHB and grown to logarithmic phase (OD_600_ of 0.5). Afterward, the culture was diluted in fresh MHB (dilution factor 1:1800) to adjust a final cell concentration of ≈10^5^ cfu mL^−1^. For PPNA treatment, 190 µL were transferred into 96 well plates followed by a preincubation step at 37 °C for 5 min and 350 rpm shaking (ThermoMixer, Eppendorf) to reach physiological temperature. Immediately, 10 µL of 20x PPNA solutions were added to adjust the following final PNA concentrations: 5  and 10 µm for JVpna‐1135/JVpna‐1138 (*murA*) or 2.5 µm for JVpna‐177 (*acpP*)/JVpna‐178 (scrambled). Water was added instead of PPNA at an equivalent volume serving as negative controls, whereas a scrambled PPNA served as sequence‐unrelated control. An aliquot of the input sample and aliquots of each condition were spotted on LB agar plates at 15 , 30 , 60 , 120 , 240 min and 24 h post treatment. For this, 5 µL of undiluted and 5 µL of serially diluted (10^−1^ to 10^−7^) samples were transferred. After each time point, the plate was placed back to 37 °C and constant shaking at 350 rpm (ThermoMixer, Eppendorf).

## Conflict of Interest

The authors declare no conflict of interest.

## Supporting information



Supporting Information

## Data Availability

The data that support the findings of this study are available from the corresponding author upon reasonable request.
