# Supplementary material for: High‐Throughput Tiling of Essential mRNAs Increases Potency of Antisense Antibiotics
Source: Adv Sci (Weinh). 2025 Apr 30;12(28):2504284. doi: 10.1002/advs.202504284 (PMC12302540; doi:10.1002/advs.202504284)

Supporting Information

**High-Throughput Tiling of Essential mRNAs**

**Increases Potency of Antisense Antibiotics**

Giorgia Danti^†^, Linda Popella^†^, Jörg Vogel^*^, Hans M. Maric*

**Outline**

**Supplementary Table 1.** Commercial PPNAs p.2

**Supplementary Table 2.** Fluorescently-labelled RNA probes p.3

**Supplementary Table 3.** Summary of MICs for the tested cPPNA versus dPPNA candidates

with their respective sequences. p.3

**Supplementary Figure 1** Microarray PNA/RNA hybridization data 10 and 26 nt template p.4

**Supplementary Figure 2** Microarray PNA/RNA hybridization data 18 nt template p.5

**Supplementary Figure 3** Microarray PNA/RNA hybridization data 80 nt template p.6

**Supplementary Figure 4** Microarray PNA/RNA hybridization data 10 and 26 nt 5’ template p.7

**Supplementary Figure 5** Optimal PNA sequences with minimal length requirements (rpsH) p.8

**Appendix** Analytical data  p.9-32

**Supplementary Table 1. Commercial PPNAs.** Sequences are shown from N- to C-termini.

| **ID** | **Cell Penetrating Peptide** | **PNA sequence** | **Target window** |  |
| --- | --- | --- | --- | --- |
| JVpna-1332 | KFFKFFKFFK | taaatttcc | -14;-6 | Peps4LS |
| JVpna-1333 | KFFKFFKFFK | ttaaatttc | -13;-5 | Peps4LS |
| JVpna-1334 | KFFKFFKFFK | cttaaattt | -12;-4 | Peps4LS |
| JVpna-1335 | KFFKFFKFFK | tcttaaatt | -11;-3 | Peps4LS |
| JVpna-1336 | KFFKFFKFFK | ctcttaaat | -10;-2 | Peps4LS |
| JVpna-1337 | KFFKFFKFFK | actcttaaa | -9;-1 | Peps4LS |
| JVpna-1338 | KFFKFFKFFK | tactcttaa | -8;1 | Peps4LS |
| JVpna-1339 | KFFKFFKFFK | atactctta | -7;2 | Peps4LS |
| JVpna-1340 | KFFKFFKFFK | catactctt | -6;3 | Peps4LS |
| JVpna-1341 | KFFKFFKFFK | tcatactct | -5;4 | Peps4LS |
| JVpna-1342 | KFFKFFKFFK | ctcatactc | -4;5 | Peps4LS |
| JVpna-1343 | KFFKFFKFFK | gctcatact | -3;6 | Peps4LS |
| JVpna-1344 | KFFKFFKFFK | tgctcatac | -2;7 | Peps4LS |
| JVpna-1345 | KFFKFFKFFK | gtgctcata | -1;8 | Peps4LS |
| JVpna-1346 | KFFKFFKFFK | agtgctcat | 1;9 | Peps4LS |
| JVpna-1347 | KFFKFFKFFK | tagtgctca | 2;10 | Peps4LS |
| JVpna-1348 | KFFKFFKFFK | atagtgctc | 3;11 | Peps4LS |
| JVpna-1349 | KFFKFFKFFK | gatagtgct | 4;12 | Peps4LS |
| JVpna-867 | KFFKFFKFFK | catctgtct | -6;3 | Peps4LS |
| JVpna-1352 | KFFKFFKFFK | tccatttag | -4;5 | Peps4LS |
| JVpna-1353 | KFFKFFKFFK | tagtttctc | -8;1 | Peps4LS |
| JVpna-177 | KFFKFFKFFK | ctcatactc | -4;5 | Peps4LS |
| JVpna-178 | KFFKFFKFFK | cactatctc | scrambled | Peps4LS |

**Supplementary Table 2. Fluorescently-labelled RNA probes**

| Name | 5’ modification | Sequence (5’-to 3’-orientation) | 3’ modification |  |
| --- | --- | --- | --- | --- |
| ^10^acpP-Cy5 | - | GAGUAUGAGC | Cy5 | GenScript |
| ^26^acpP-Cy5, | - | GGAAAUUUAAGAGUAUGAGCACUAUC | Cy5 | GenScript |
| ^80^acpP-Cy5 | - | AACCAUCGCGAAAGCGAGUUUUGAUAGGAAAUUUAAGAGUAUGAGCACUAUCGAAGAACGCGUUAAGAAAAUUAUCGGCG | Cy5 | Eurofins |
| ^10^rpsH-Cy3 | - | ACAGAUGAGC | Cy3 | GenScript |
| ^26^rpsH-Cy3 | - | GGGAGUAAAGACAGAUGAGCAUGCAA | Cy3 | GenScript |
| ^80^rpsH-Cy3 | - | CUGGUAAUUGUCACCAAUUGAAUCACGGGAGUAAAGACAGAUGAGCAUGCAAGAUCCGAUCGCGGAUAUGCUGACCCGUA | Cy3 | Eurofins |
| Cy5-^10^acpP | Cy5 | GAGUAUGAGC |  | GenScript |
| Cy5-^26^acpP | Cy5 | GGAAAUUUAAGAGUAUGAGCACUAUC |  | GenScript |

**Supplementary Table 3: Summary of MICs for the tested cPPNA versus dPPNA candidates with their respective sequences.**

| **PNA#**  **(cPPNA)** | **MIC**  **(cPPNA)** | **PNA#**  **(dPPNA)** | **MIC (dPPNA)** | **Sequence** | **Target** |
| --- | --- | --- | --- | --- | --- |
| **^JVpna867^** | **^2.5-5 µM*^** | **^JVpna1105^** | **^10-20 µM^** | **^catctgtct^** | **^rpsH^** |
| **^JVpna1352^** | **^2.5 µM^** | **^JVpna1138^** | **^5 µM^** | **^tccatttag^** | **^murA^** |
| **^JVpna1353^** | **^2.5-5 µM**^** | **^JVpna1145^** | **^5 µM^** | **^tagtttctc^** | **^ftsZ^** |
| **^Jvpna177^** | **^1.25 µM^** | **^JVpna1072^** | **^5 µM^** | **^ctcatactc^** | **^acpP^** |
| **^Jvpna178^** | **^> 10 µM^** | **^JVpna1763^** | **^> 20 µM^** | **^cactatctc^** | **^scrambled^** |

*complete growth inhibition at 2.5 µM until 17 h post treatment; ** two out of four replicates showed growth at 2.5 µM with onset of growth from 16 h post treatment

**Supplementary Figure 1** Microarray PNA/RNA hybridization data for 10 nt and 26 nt templates (3’-labelled)


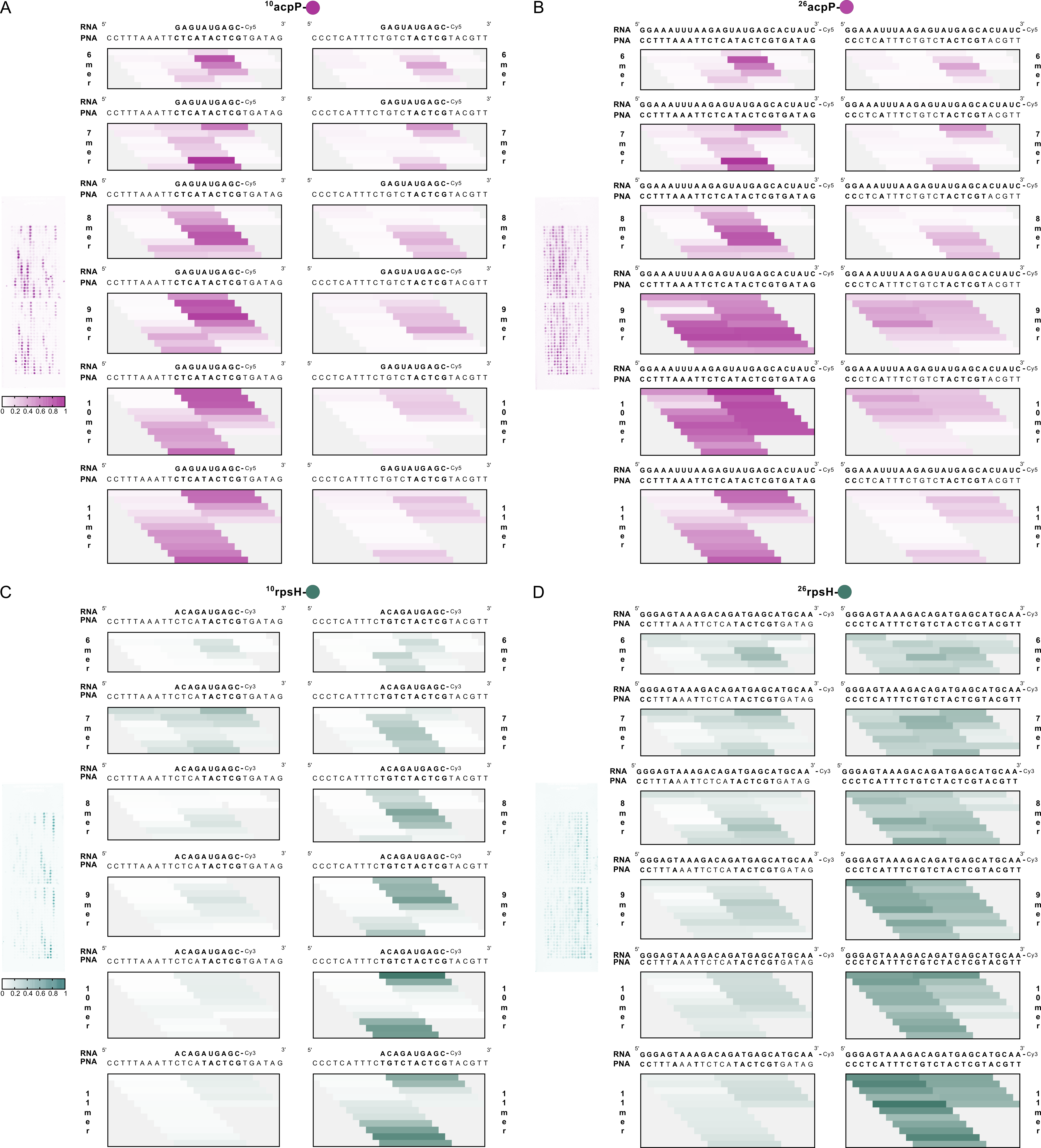


Supplementary Figure 1 (Figure S1). Microarray PNA/RNA hybridization study using 10 nt and 26 nt template RNA probes. Microarray slides with immobilized *acpP* and *rpsH* PNA sequences are incubated with fluorescently 3’-labelled template RNA probes and then imaged for binding strength quantification. Simultaneous incubation with differently labelled *acpP-* or *rpsH-*specific RNA probes mirrors binding competition and specificity. Left panels show binding strengths of the RNA probes to *acpP* PNAs, right panels show binding strengths of the RNA probes to *rpsH* PNAs. A. *acpP*-specific hybridization outcome for 10 bps long 3’-Cy5-labelled RNA probe in presence of the respective Cy3-labelled *rpsH* probe. Hybridization strength is illustrated in white-to-magenta heatmaps. B. *acpP*-specific hybridization outcome for 26 bps long 3’-Cy5-labelled RNA probe in presence of the respective Cy3-labelled *rpsH* probe. Hybridization strength is illustrated in white-to-magenta heatmaps. C. *rpsH*-specific hybridization outcome for 26 bps long *rpsH*-derived 3’-Cy3-labelled RNA probe, in presence of the respective Cy5-labelled *acpP* probe. Hybridization strength is illustrated in white-to-cyan heatmaps. Each microarray displays the library in duplicate. D. *rpsH*-specific hybridization outcome for 10 bps long *rpsH*-derived 3’-Cy3-labelled RNA probe, in presence of the respective Cy5-labelled *acpP* probe. Hybridization strength is illustrated in white-to-cyan heatmaps. Each microarray displays the library in duplicate.

**Supplementary Figure 2** Microarray PNA/RNA hybridization data 18 nt template (3’-labelled)


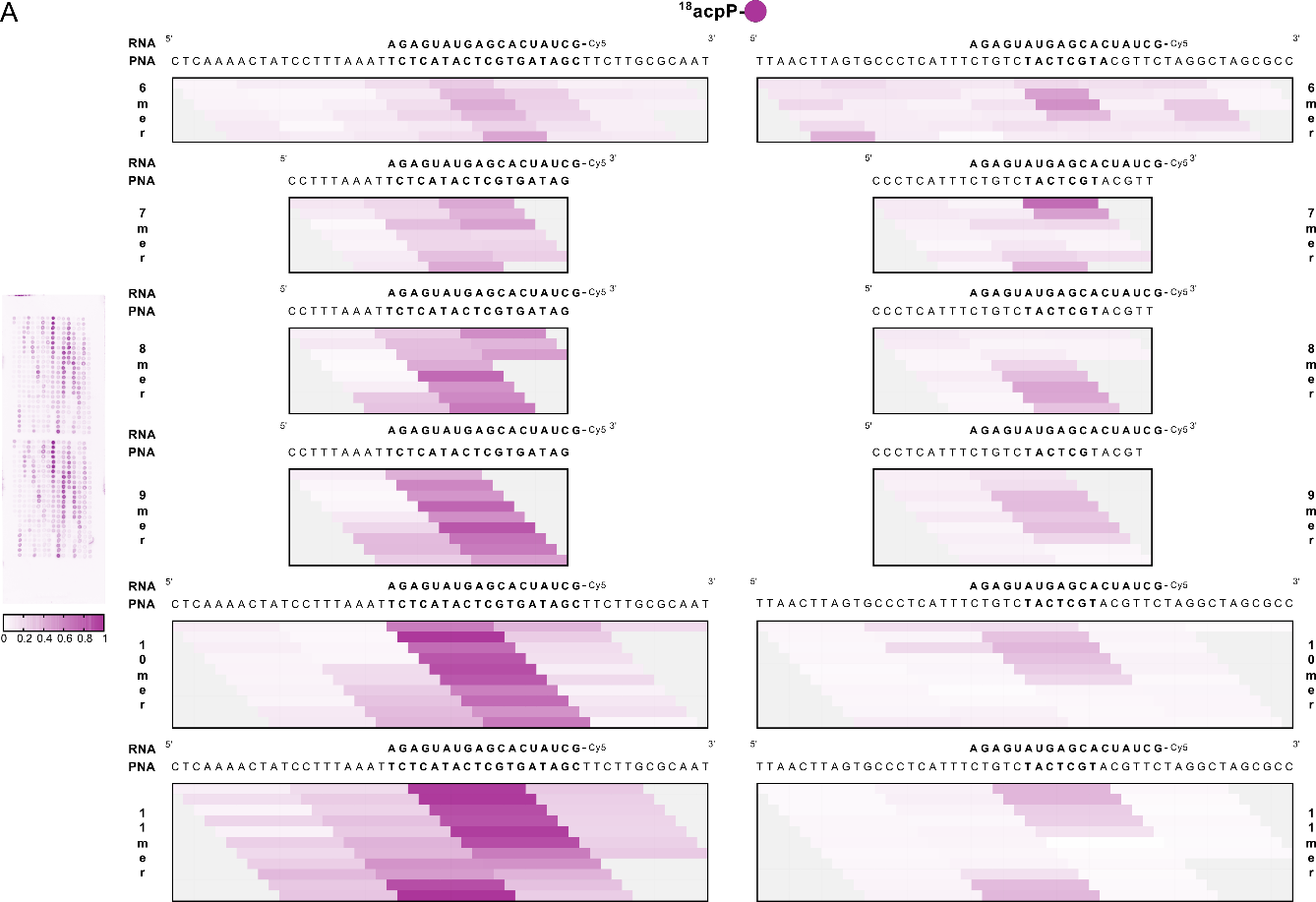


Supplementary Figure 2 (Figure S2). Microarray PNA/RNA hybridization study using 18 bps template RNA probe. A. Microarray slides with immobilized *acpP* PNA sequences are incubated with fluorescently 3’-labelled template RNA probe (18 bps) and then imaged for binding strength quantification. Hybridization strength is illustrated in white-to-magenta heatmaps. Each microarray displays the library in duplicate.

Supplementary Figure 3 Microarray PNA/RNA hybridization data 80 nt template (3’-labelled)
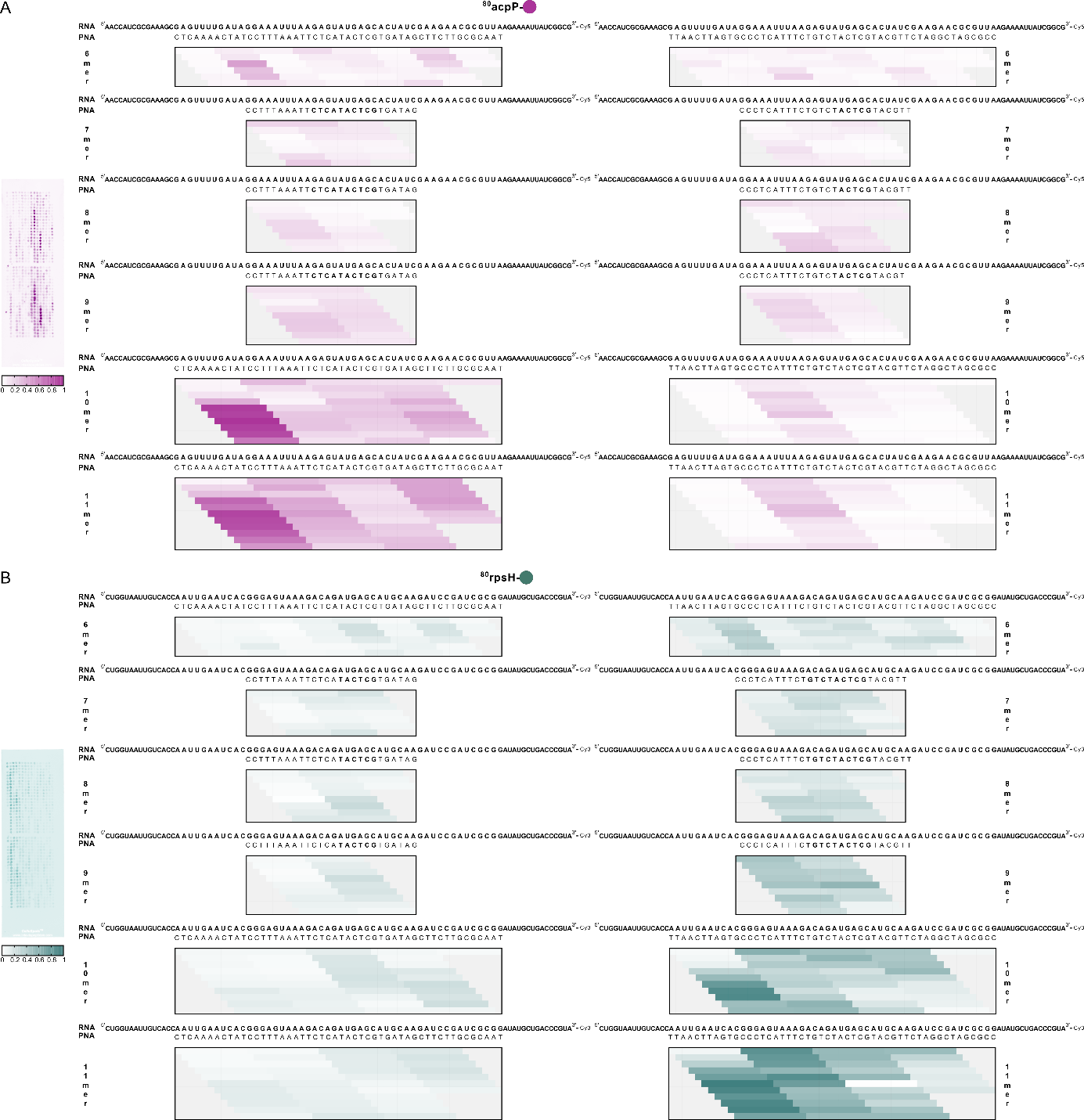


Supplementary Figure 3 (Figure S3). Microarray PNA/RNA hybridization study using 80 bps template RNA probe. A. Microarray slides with immobilized *acpP* and *rpsH* PNA sequences are incubated with fluorescently 3’-labelled template RNA probes and then imaged for binding strength quantification. Simultaneous incubation with differently labelled *acpP-* or *rpsH-*specific RNA probes mirrors binding competition and specificity. Left panels show binding strengths of the RNA probes to *acpP* PNAs, right panels show binding strengths of the RNA probes to *rpsH* PNAs. A. *acpP*-specific hybridization outcome for 80bps long 3’-Cy5-labelled RNA probe in presence of the respective Cy3-labelled *rpsH* probe. Hybridization strength is illustrated in white-to-magenta heatmaps. B. *rpsH*-specific hybridization outcome for 80bps long *rpsH*-derived 3’-Cy3-labelled RNA probe, in presence of the respective Cy5-labelled *acpP* probe. Hybridization strength is illustrated in white-to-cyan heatmaps. Each microarray displays the library in duplicate.

Supplementary Figure 4 Microarray PNA/RNA hybridization data 10 nt and 26 nt templates (5’-labelled)
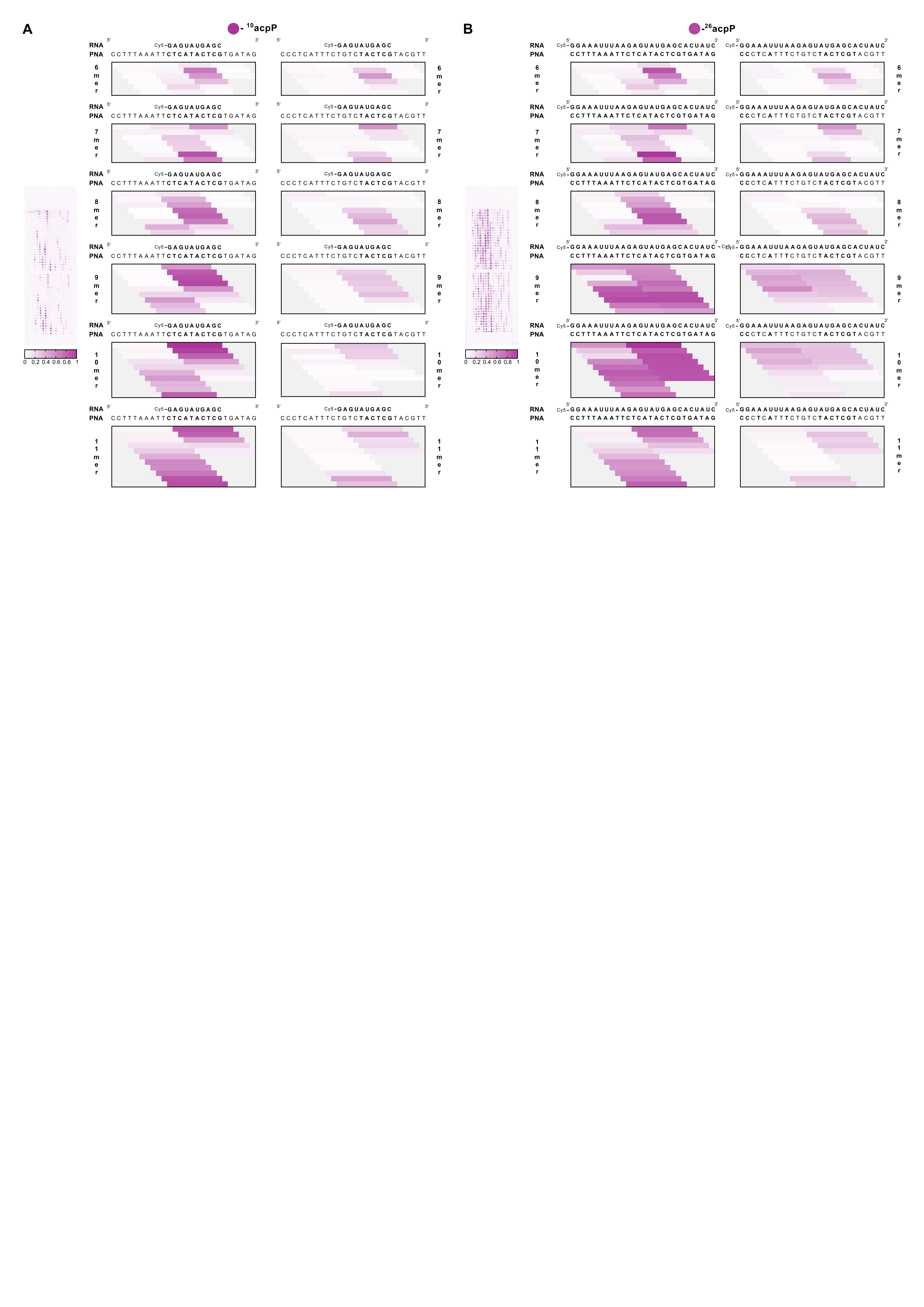


Supplementary Figure 4 (Figure S4). Microarray PNA/RNA hybridization study using 10 nt and 26 nt template RNA probes. Microarray slides with immobilized *acpP* PNA sequences are incubated with fluorescently 5’-labelled template RNA probes A. 10bps and B. 26bps, then imaged for binding strength quantification. Hybridization strength is illustrated in white-to-magenta heatmaps. Each microarray displays the library in duplicate. The experiments were performed two times independently and the average normalized binding strengths are shown. n=4

Supplementary Figure 5 Optimal PNA sequences with minimal length requirements (*rpsH*)


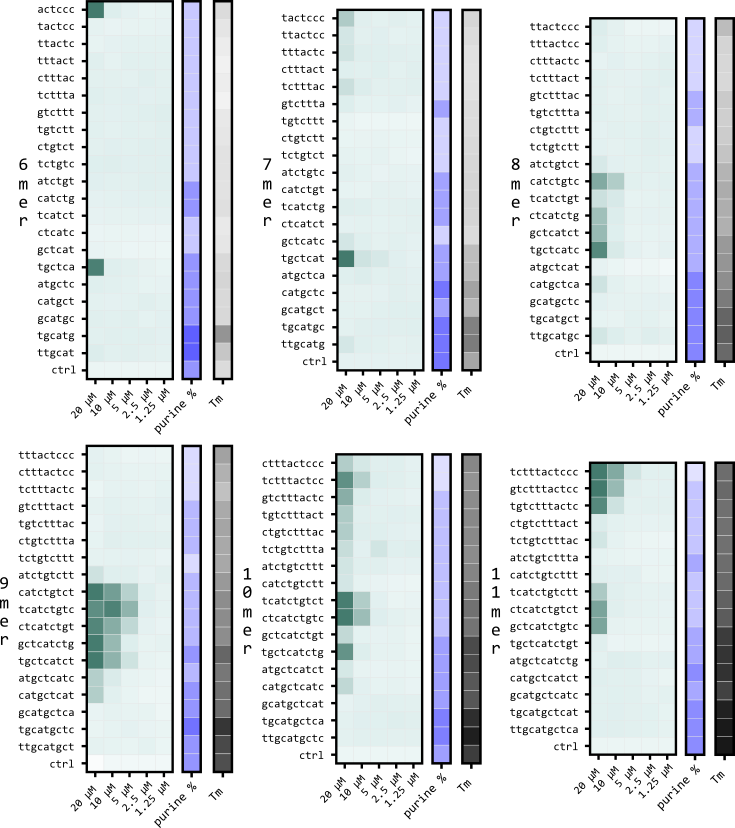


Supplementary Figure 5 (Figure S5). Identification of optimal PNA sequences with minimal length requirements (*rpsH*). Direct-to-biology validation of 6-11mer PNA sequences complementary to the *rpsH* target mRNA against UPEC 536 used at 10^5 cfu/mL in Mueller Hinton broth. Magenta-white heatmaps show the final optical density (OD_600_) at 24 h post treatment together with each PNA’s purine content (blue-scale) and its predicted metling temperature (grey-scale; <https://www.pnabio.com/support/PNA_Tool.htm>). The lowest concentration of a PPNA that inhibits growth, i.e. with a final OD_600_ of ‘<0.05’ (indicated in magenta), indicates the minimal inhibitory concentration (MIC). Scrambled PPNA controls (2^nd^ last rows per panel) were included for each set of PPNAs. MIC assays were performed at least 2 times and the average OD_600_ is shown.

Appendix

Analytical Data


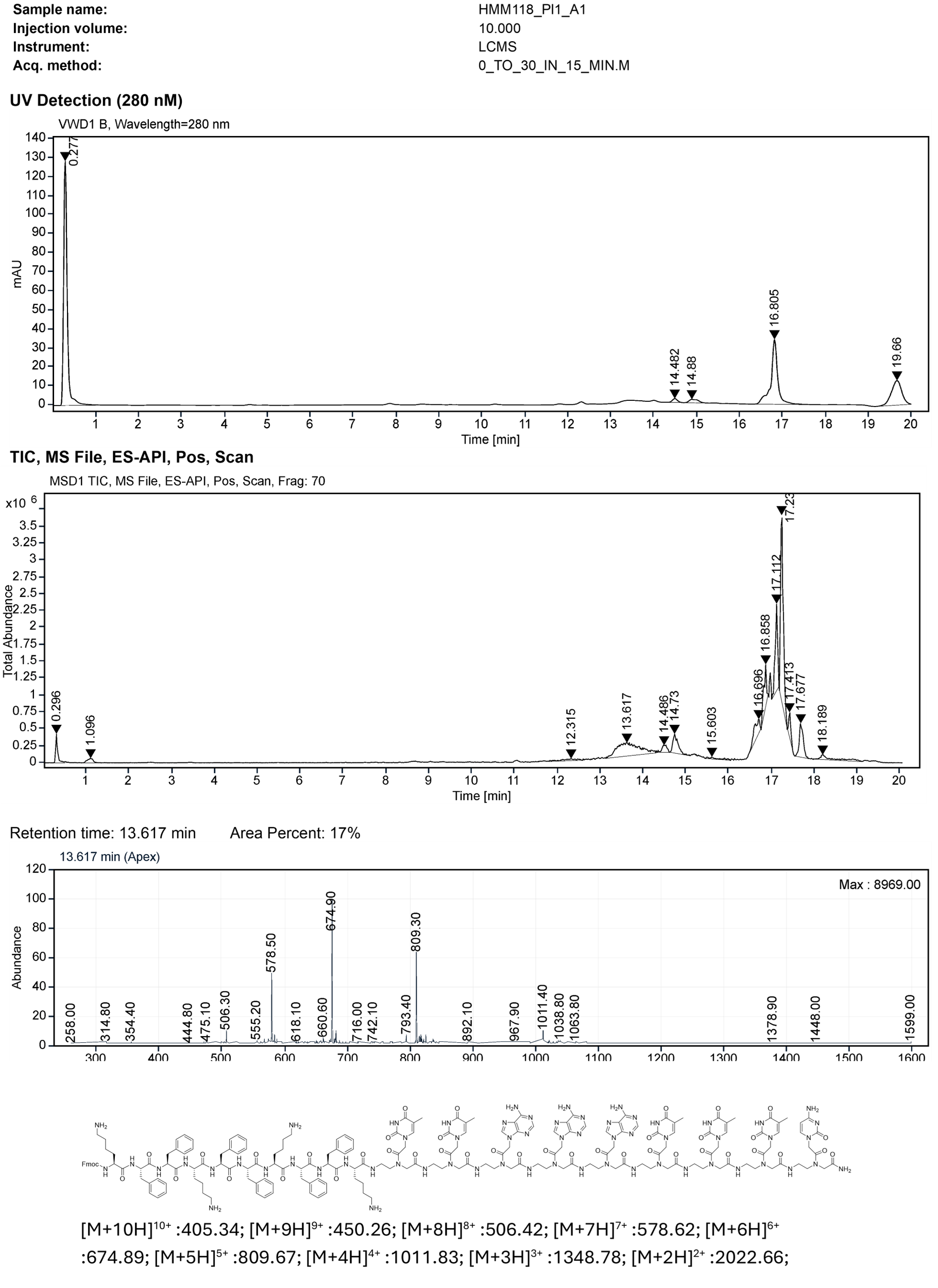

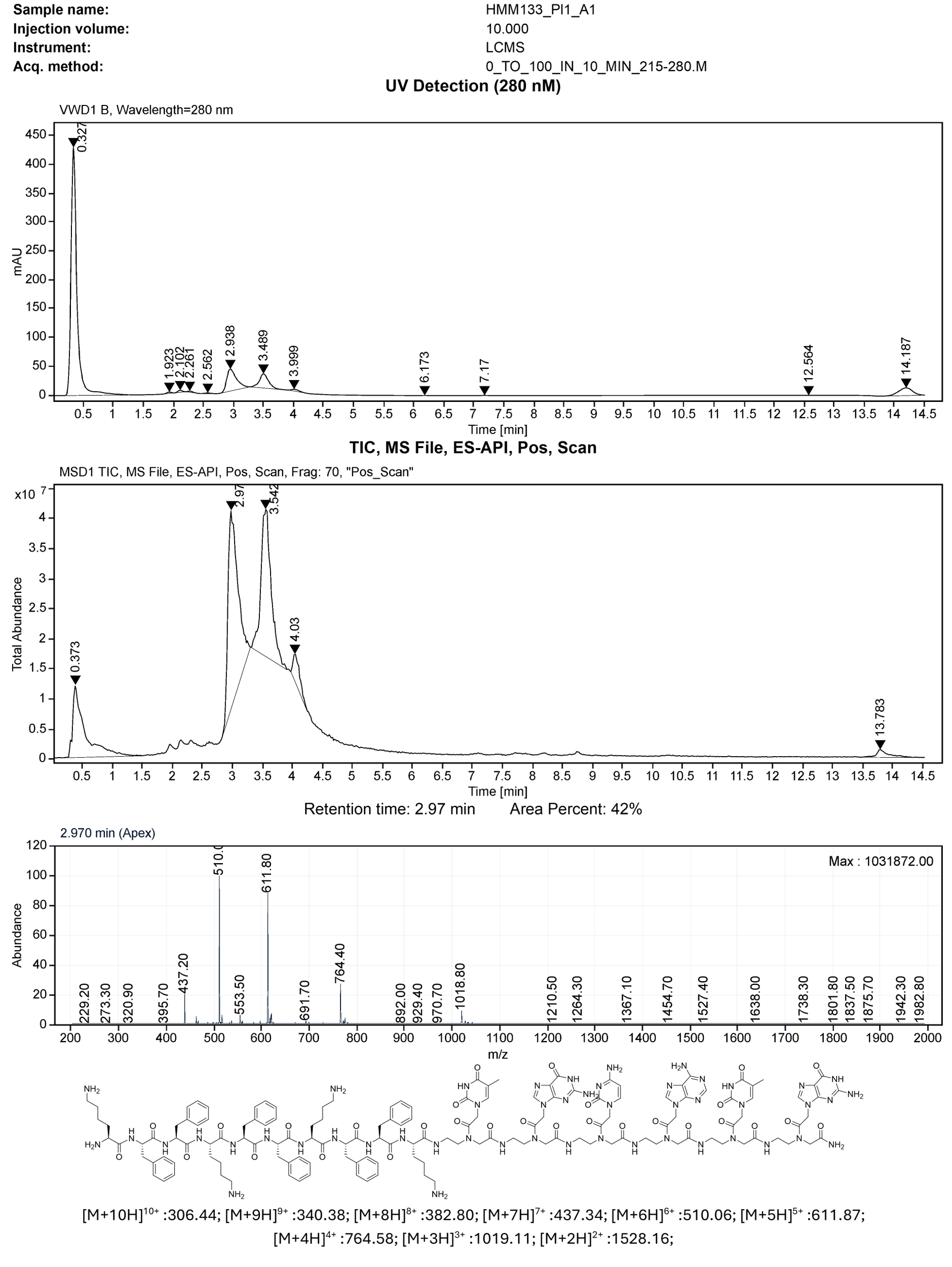

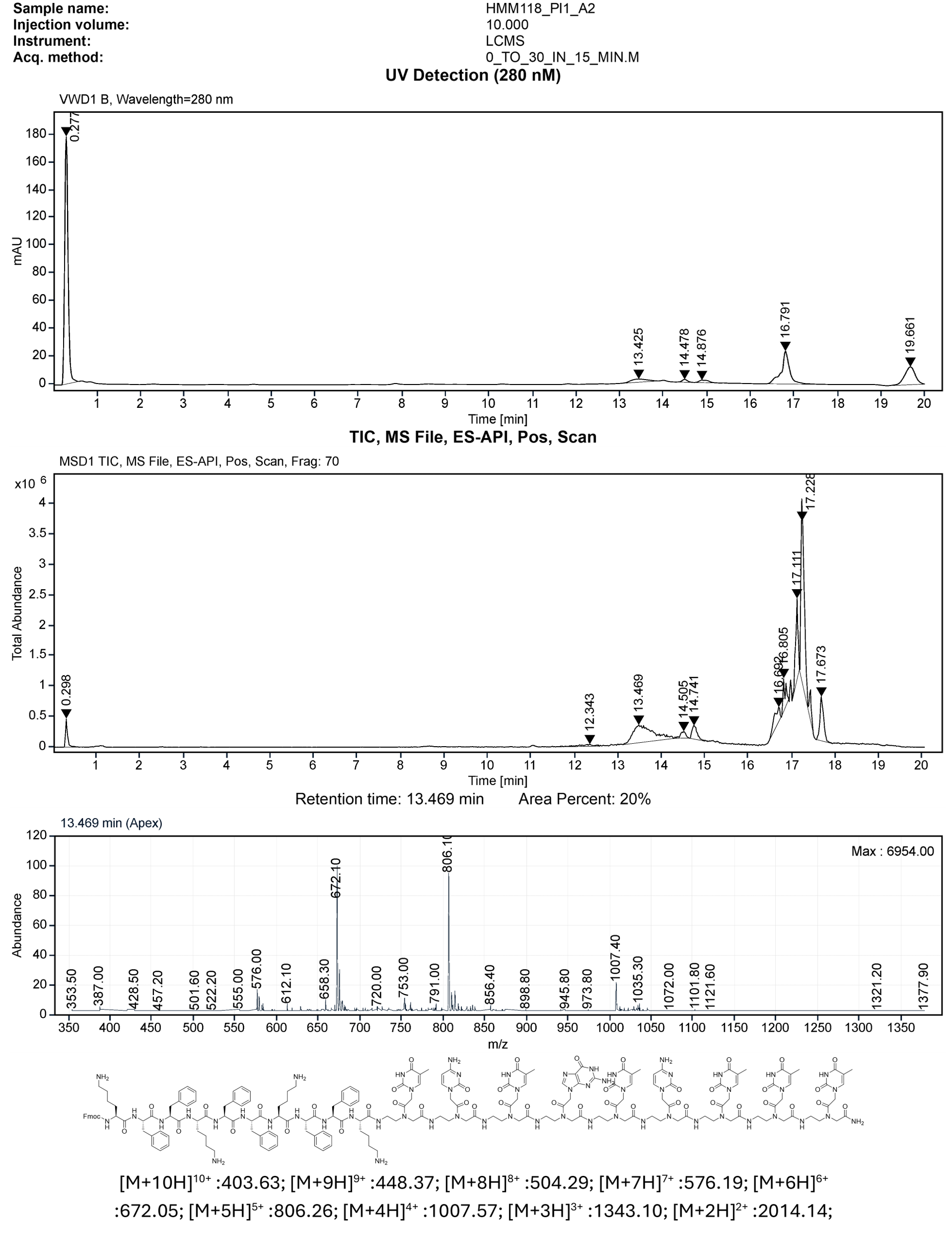

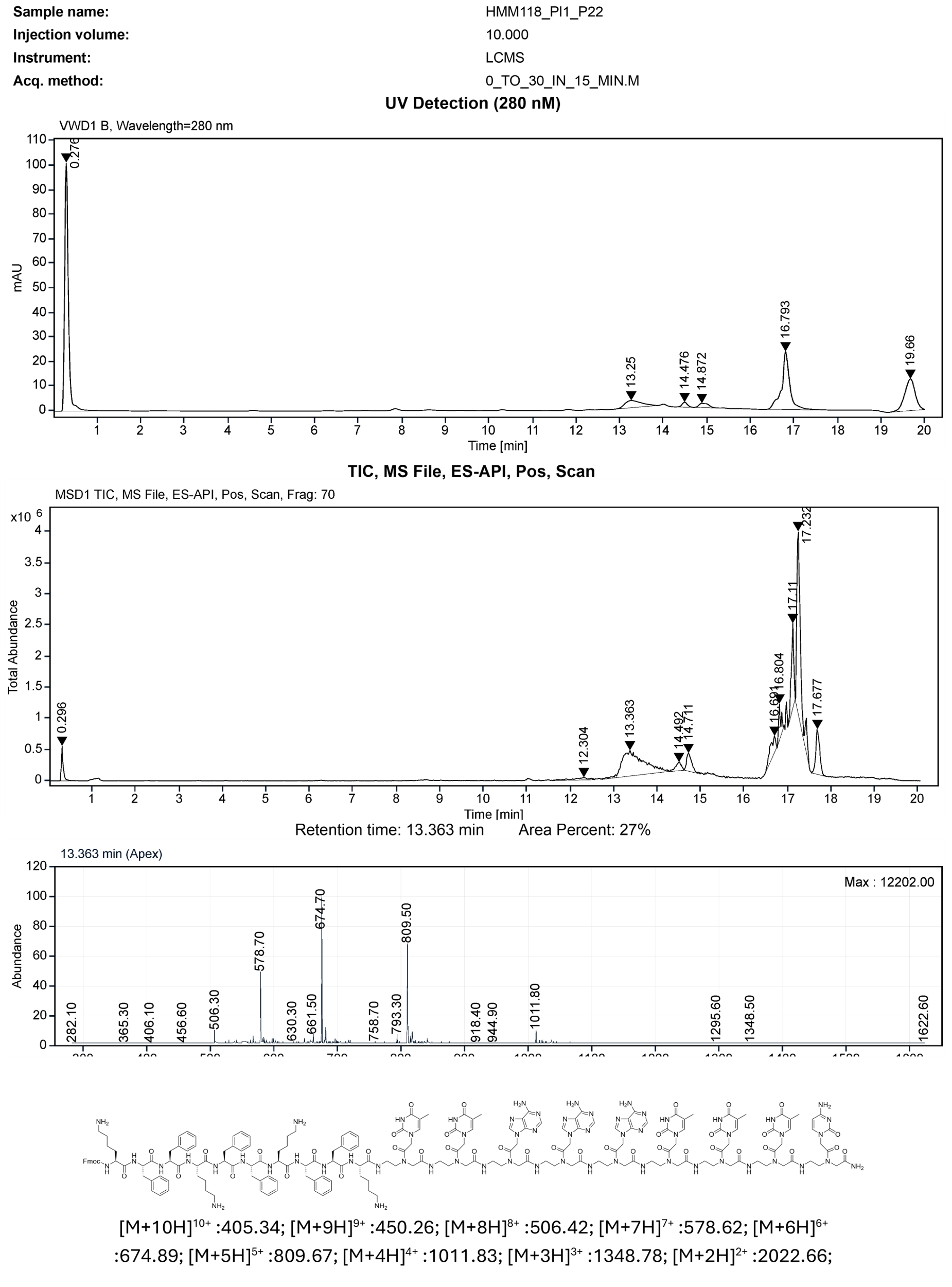

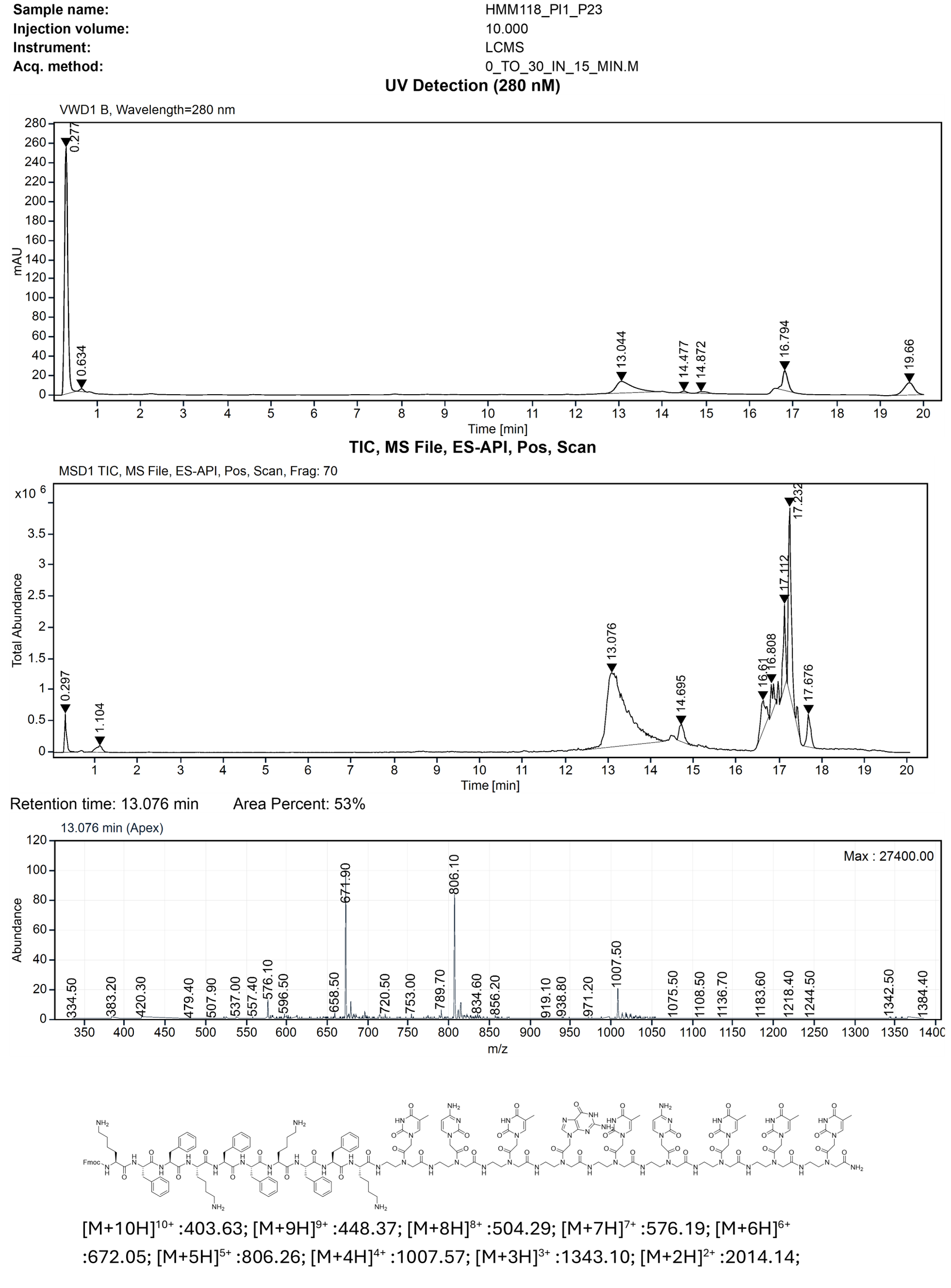

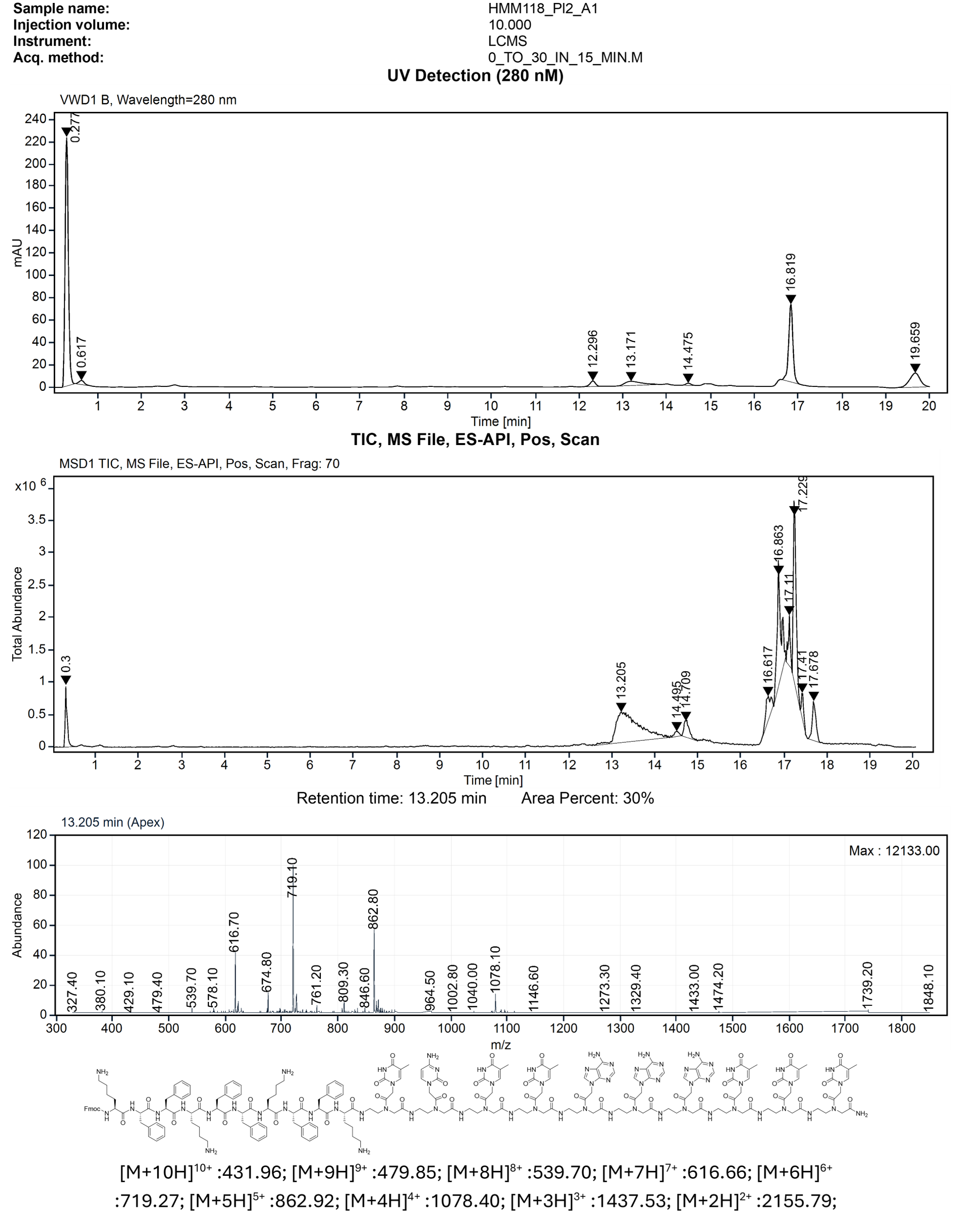

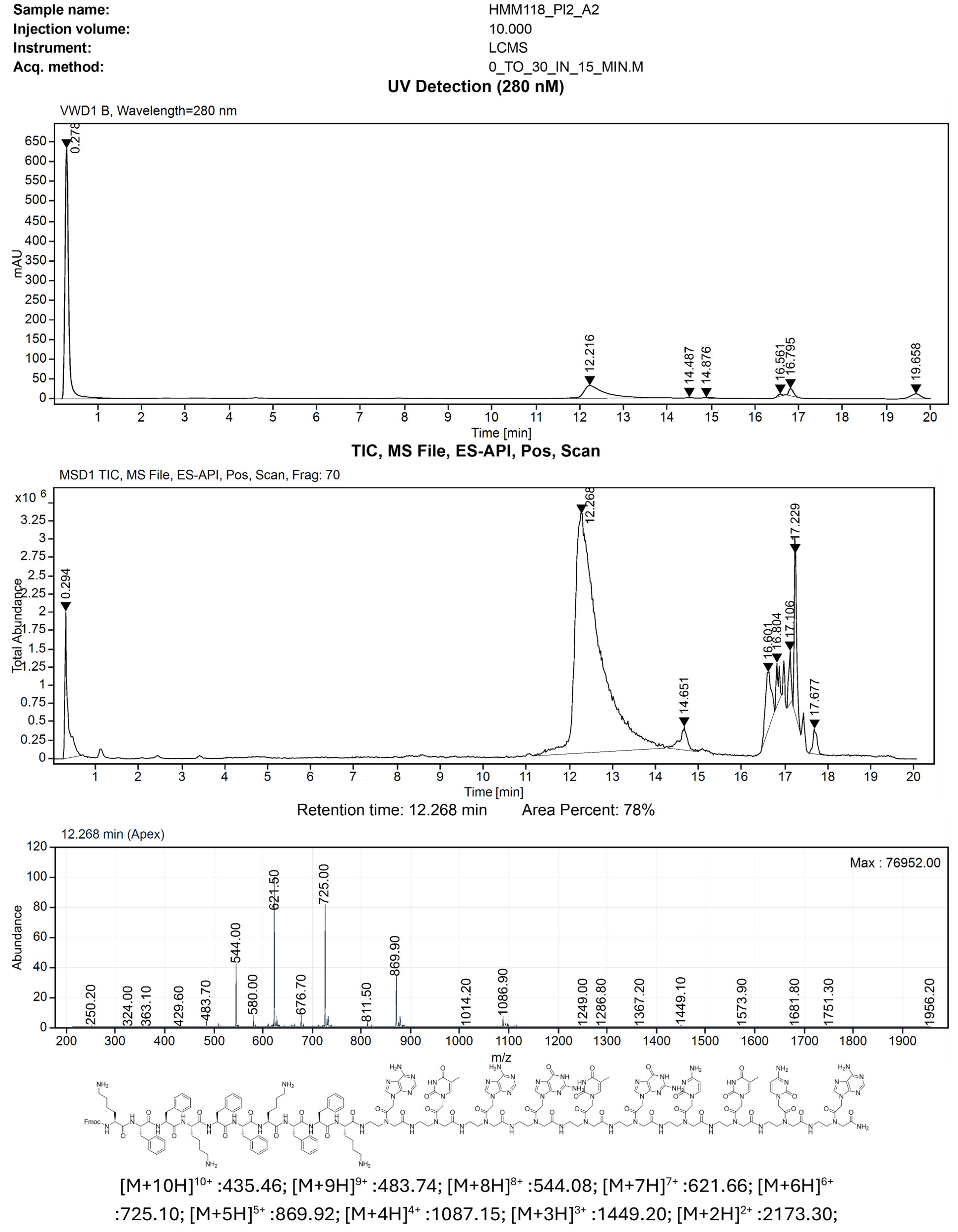

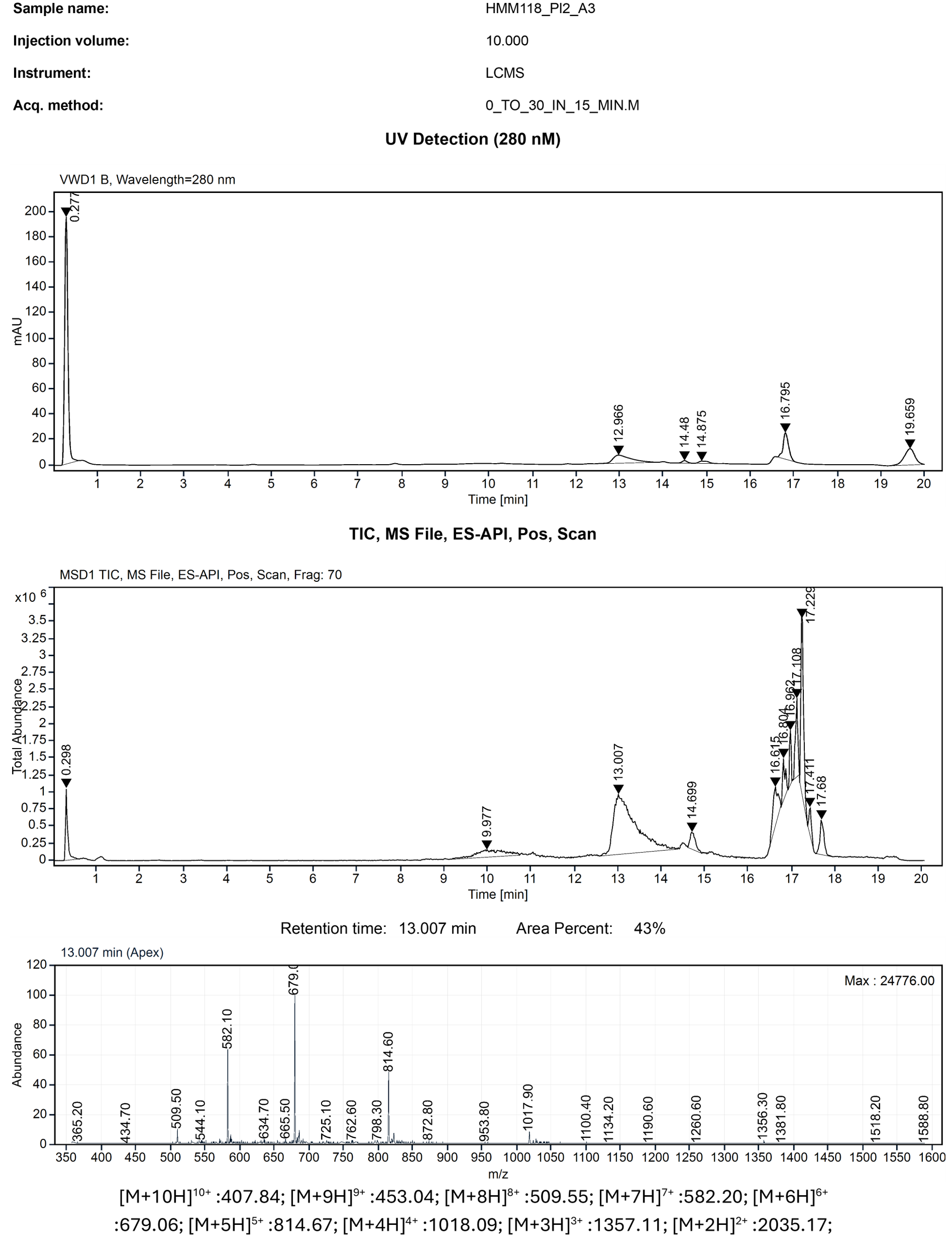

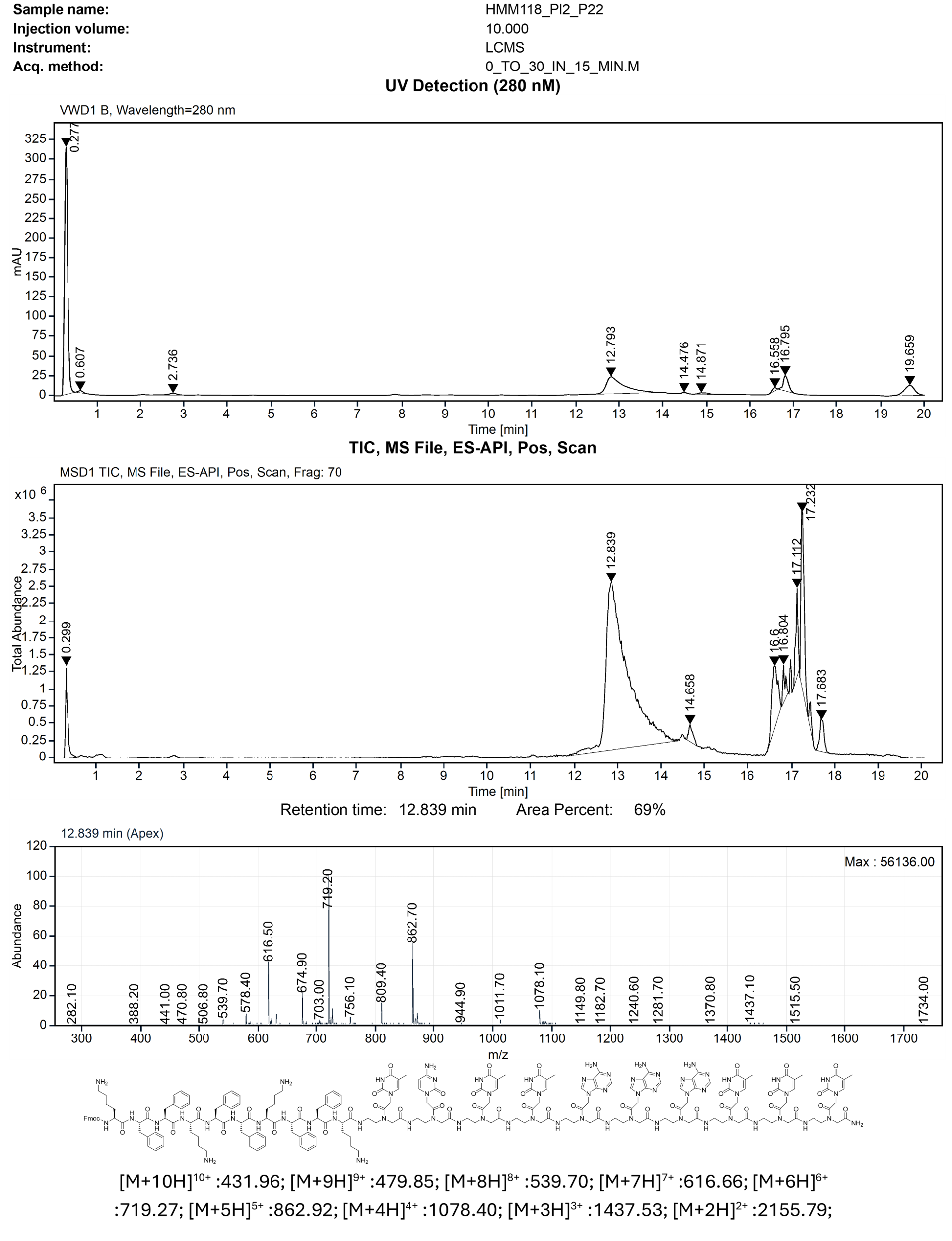

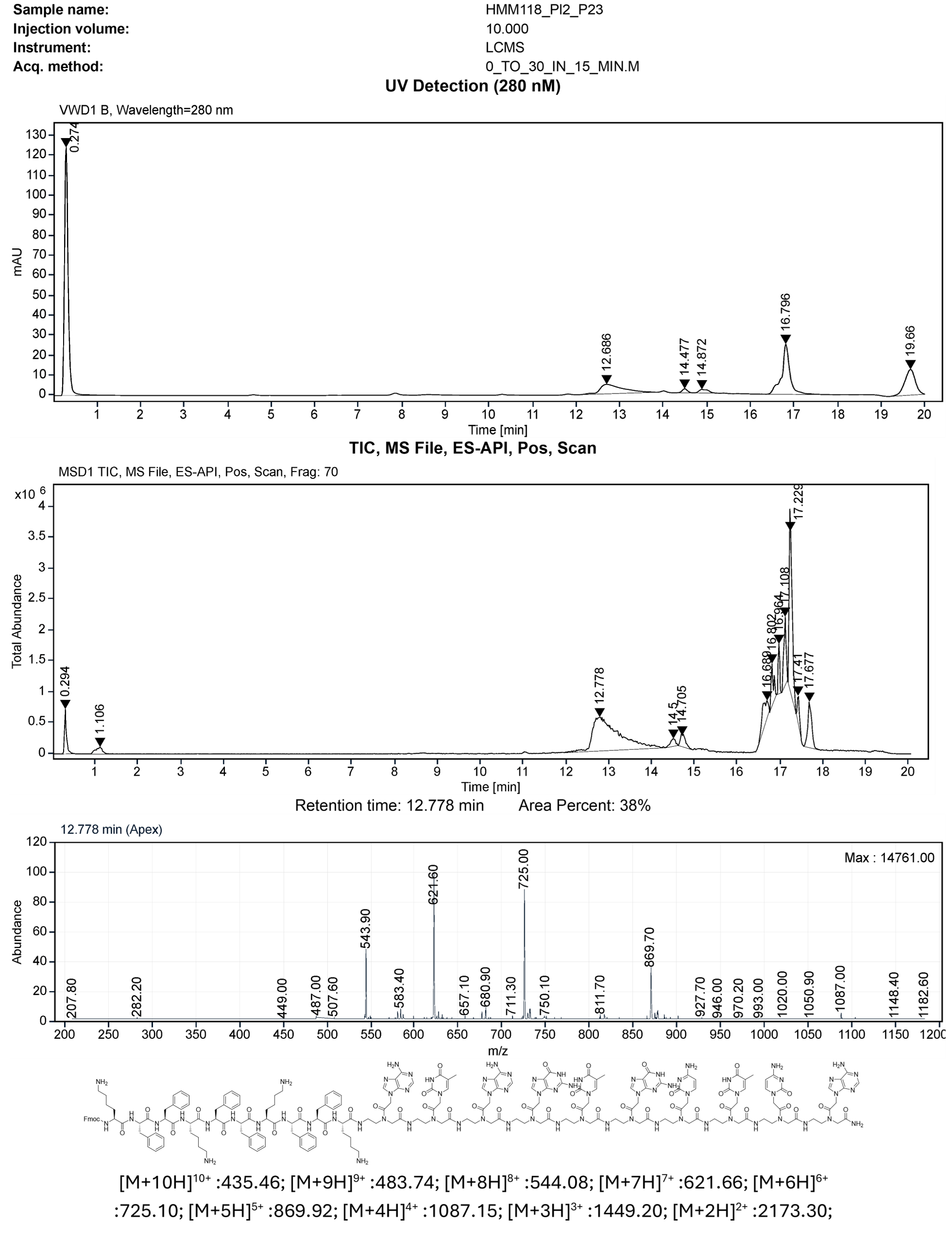

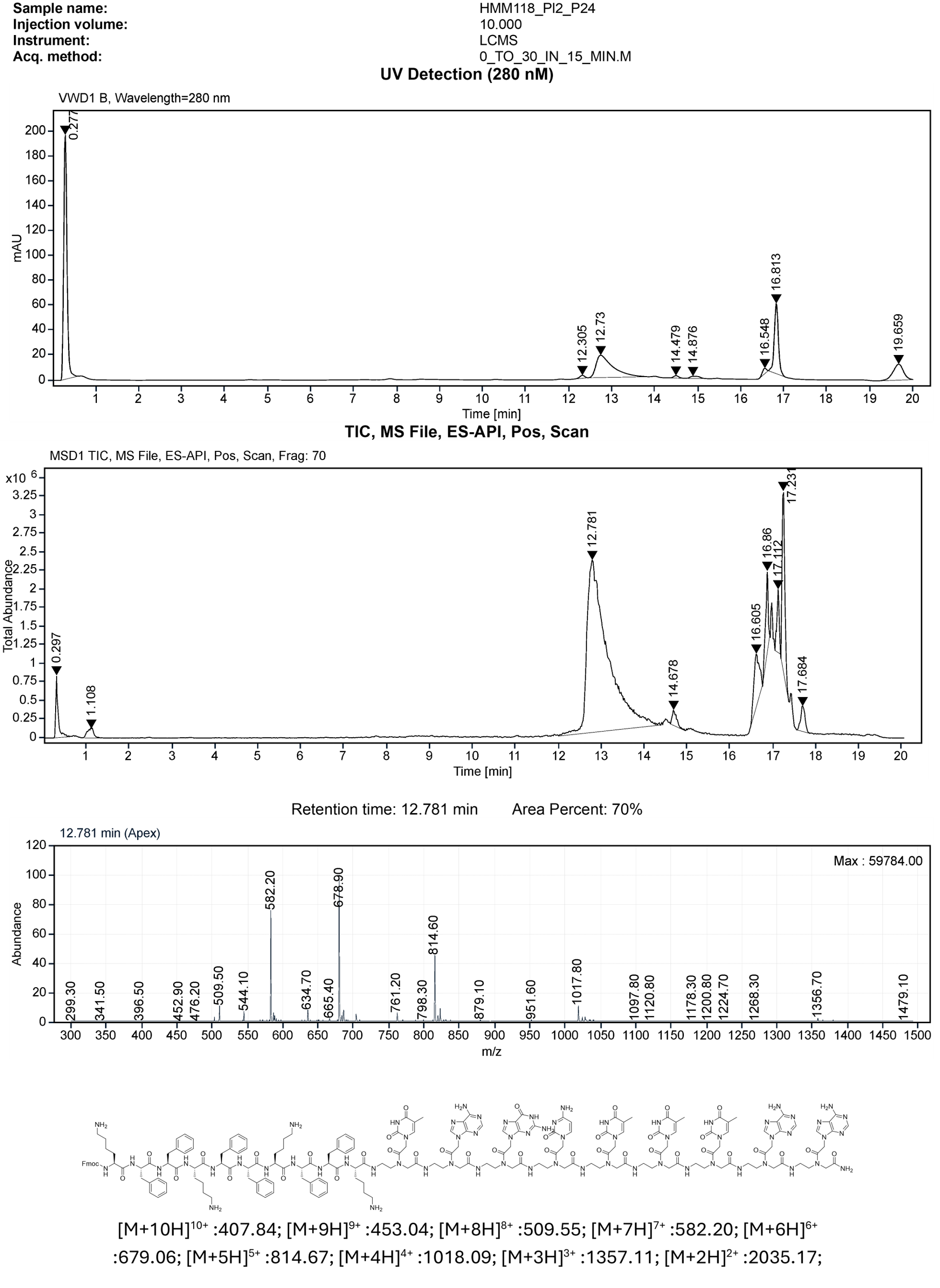

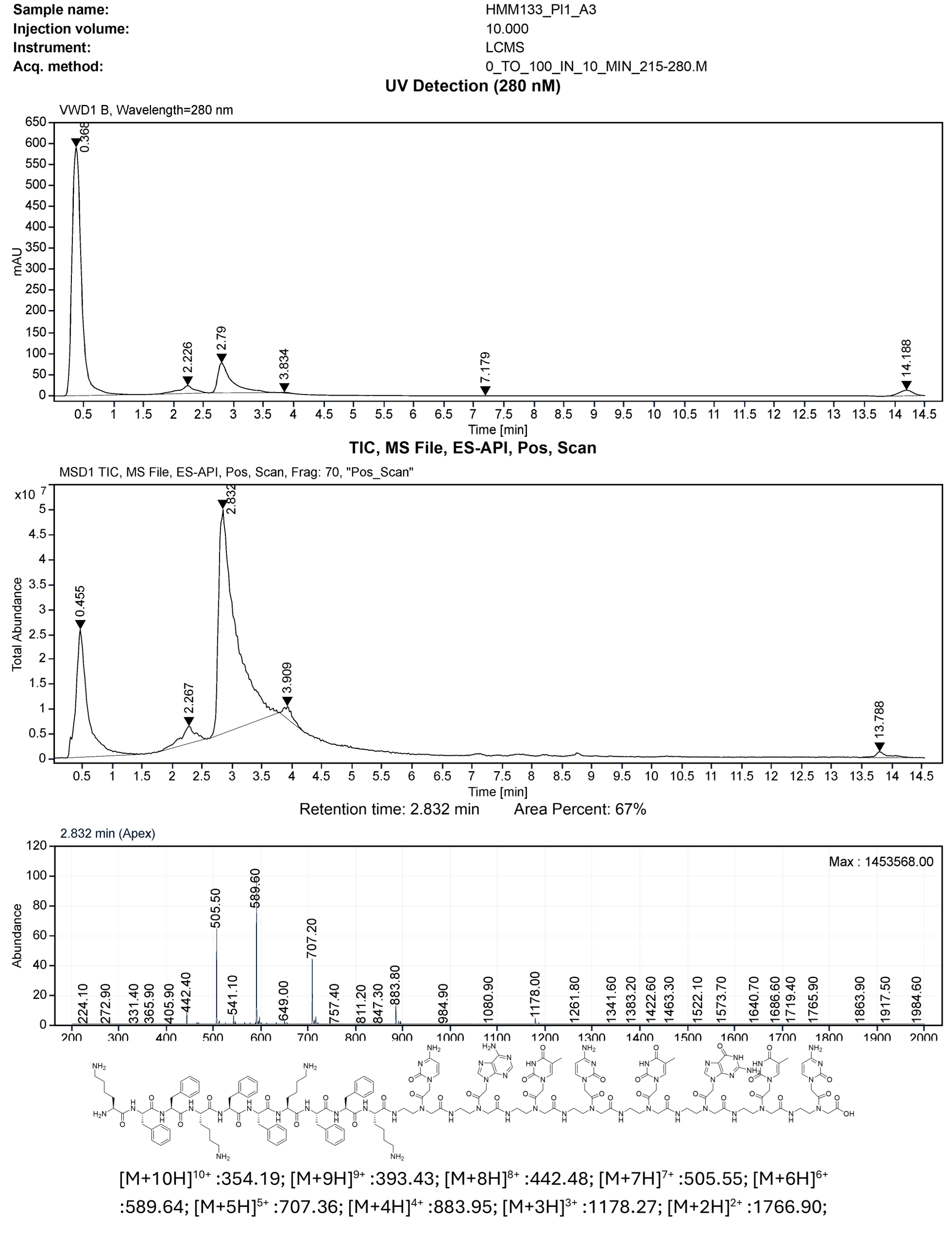

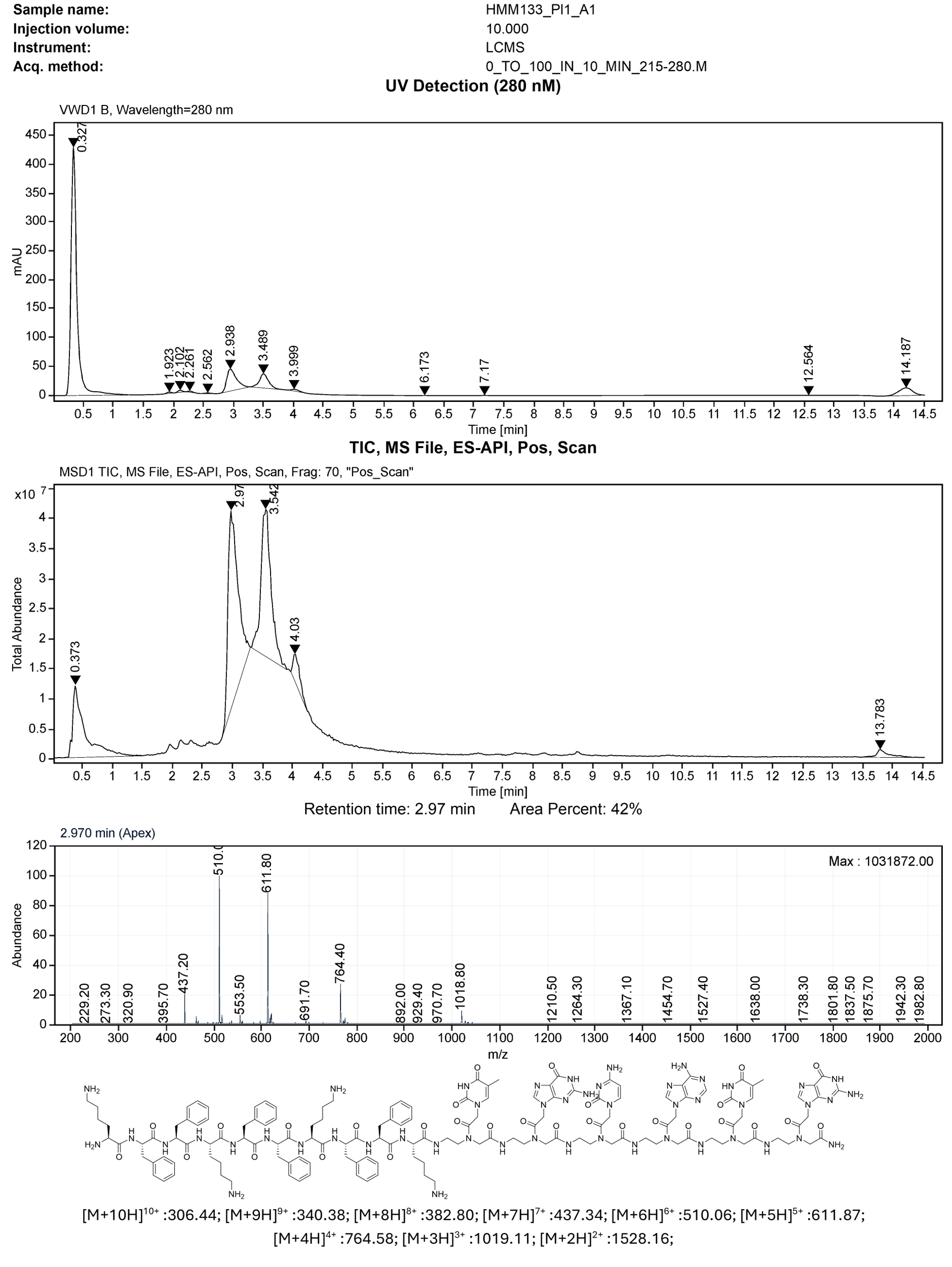

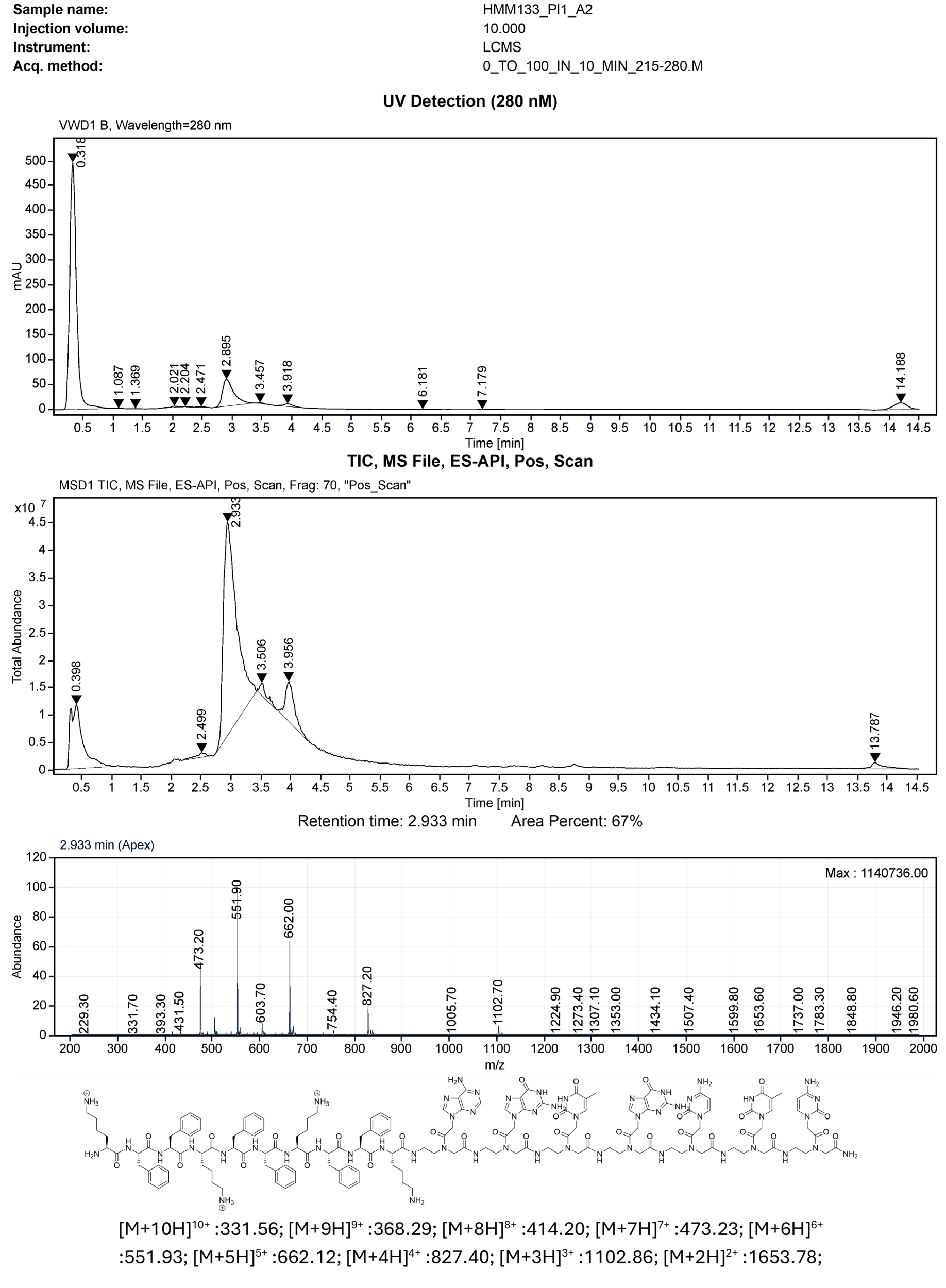

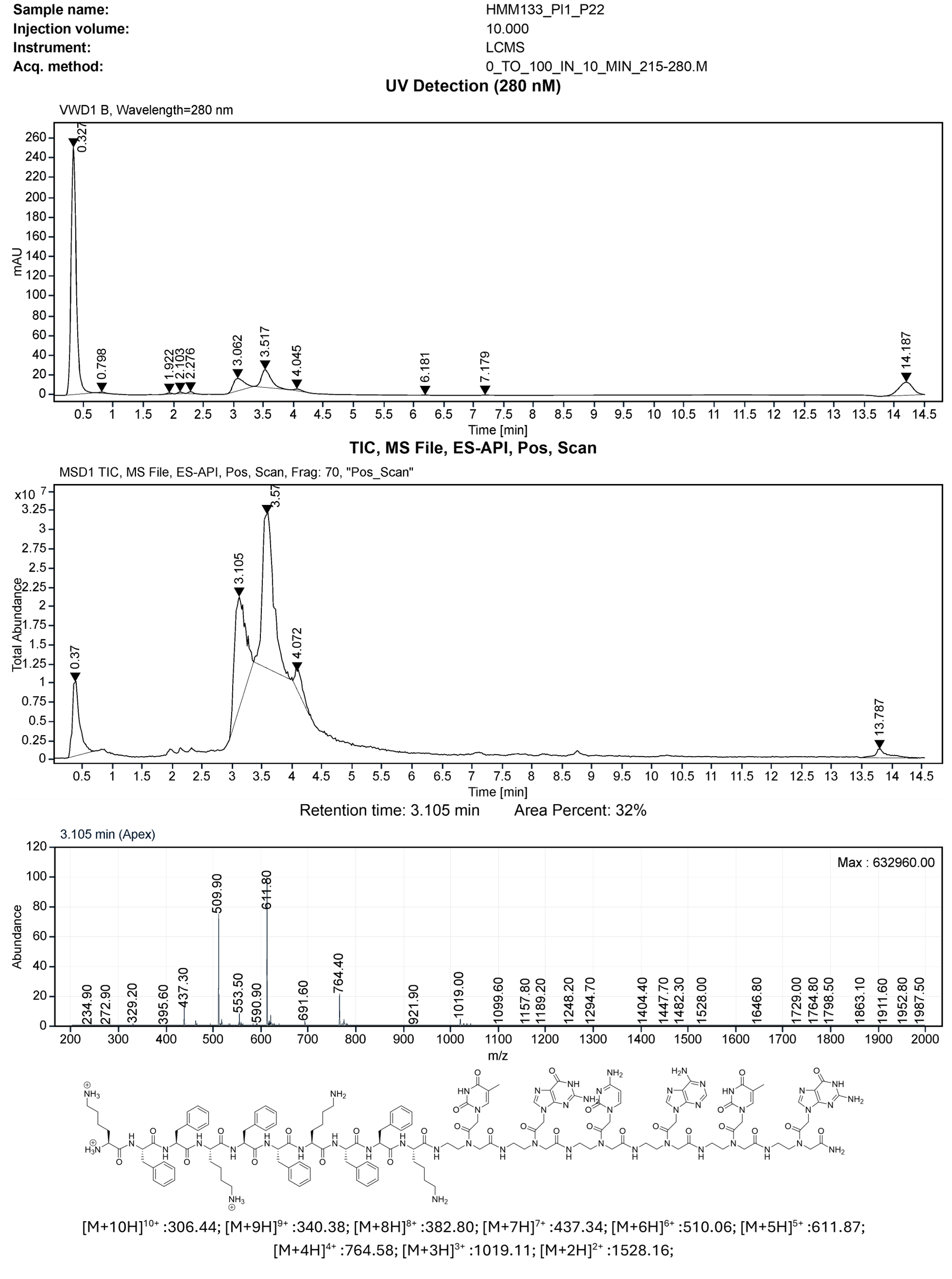

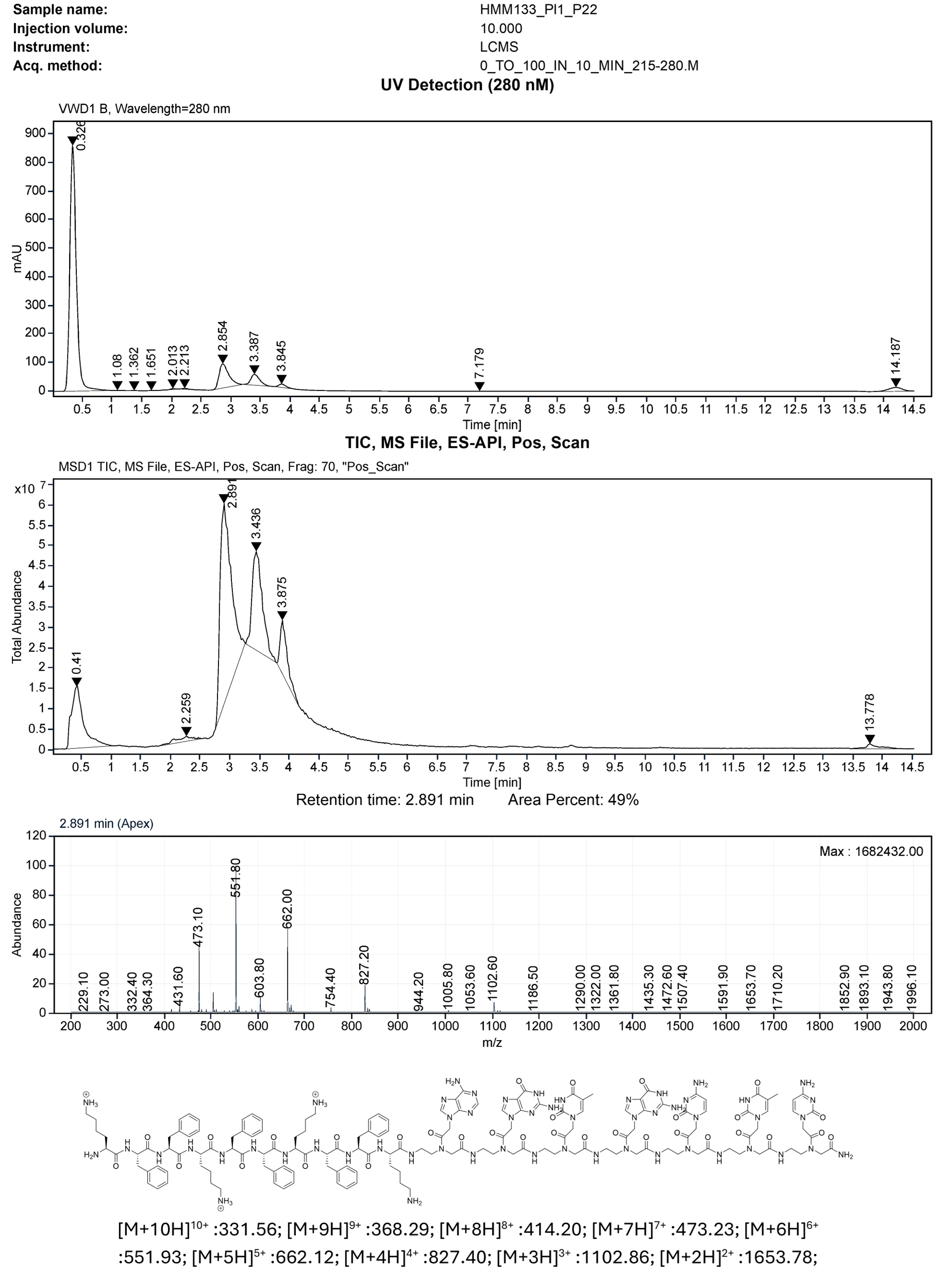

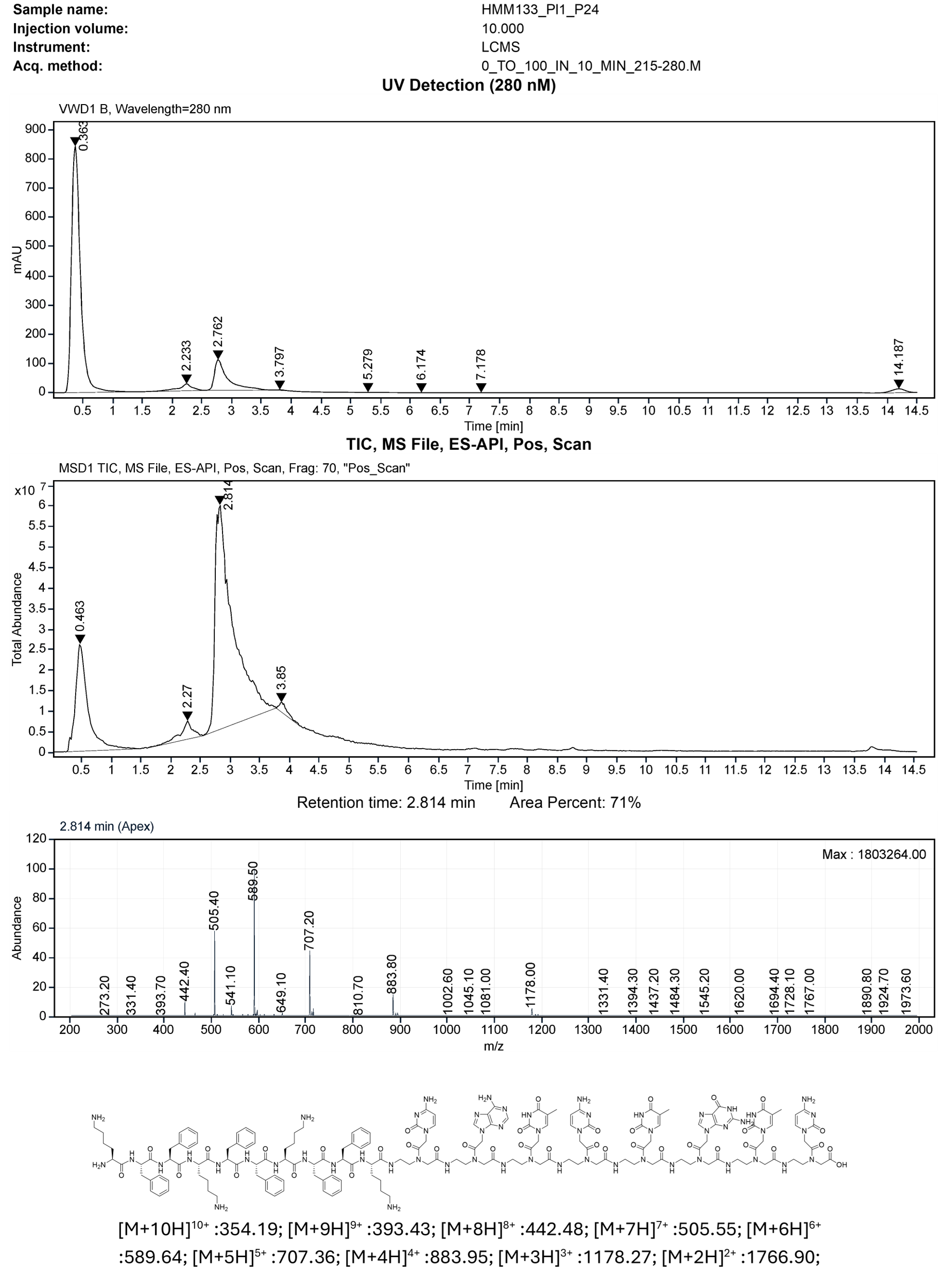

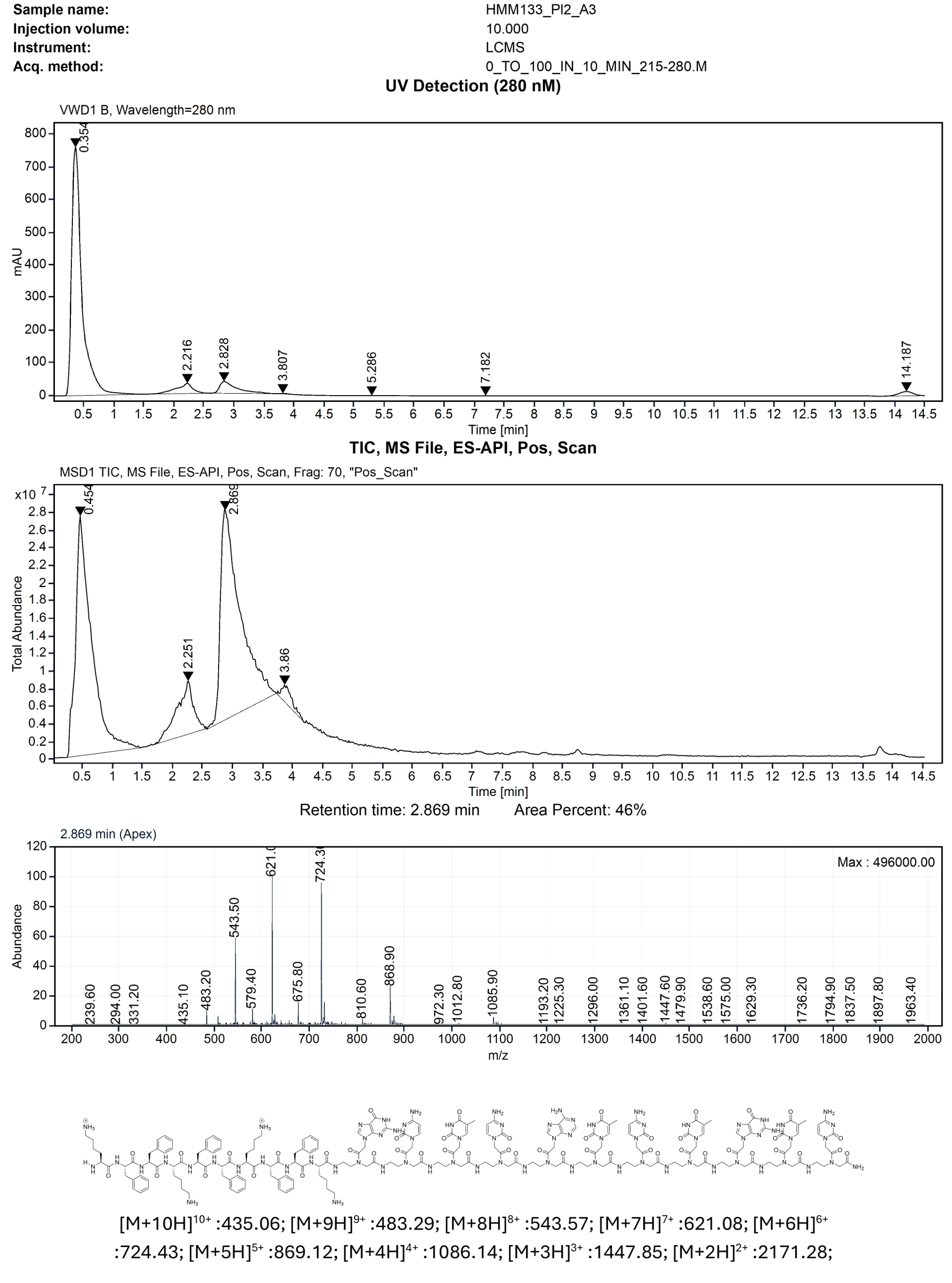

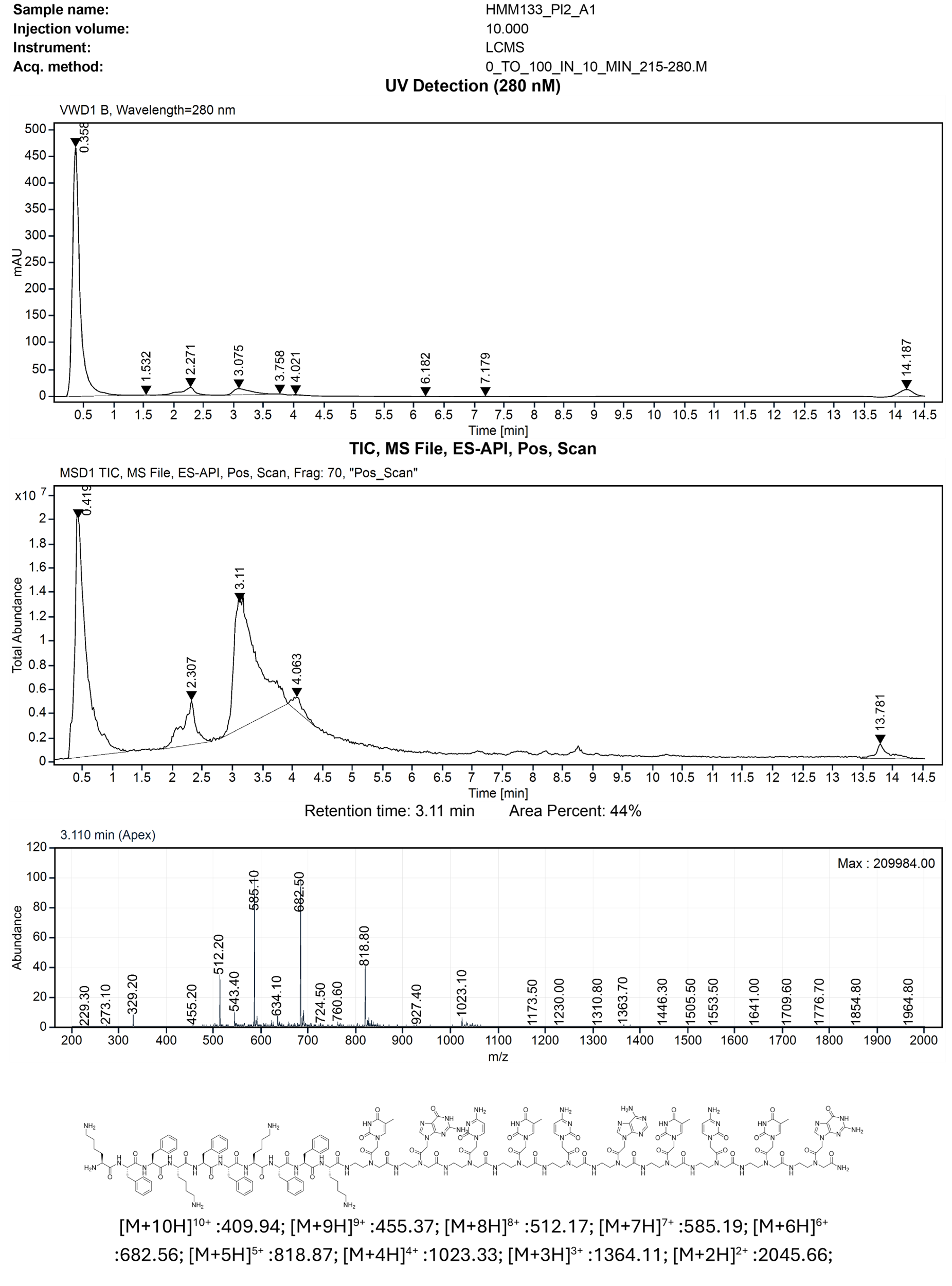

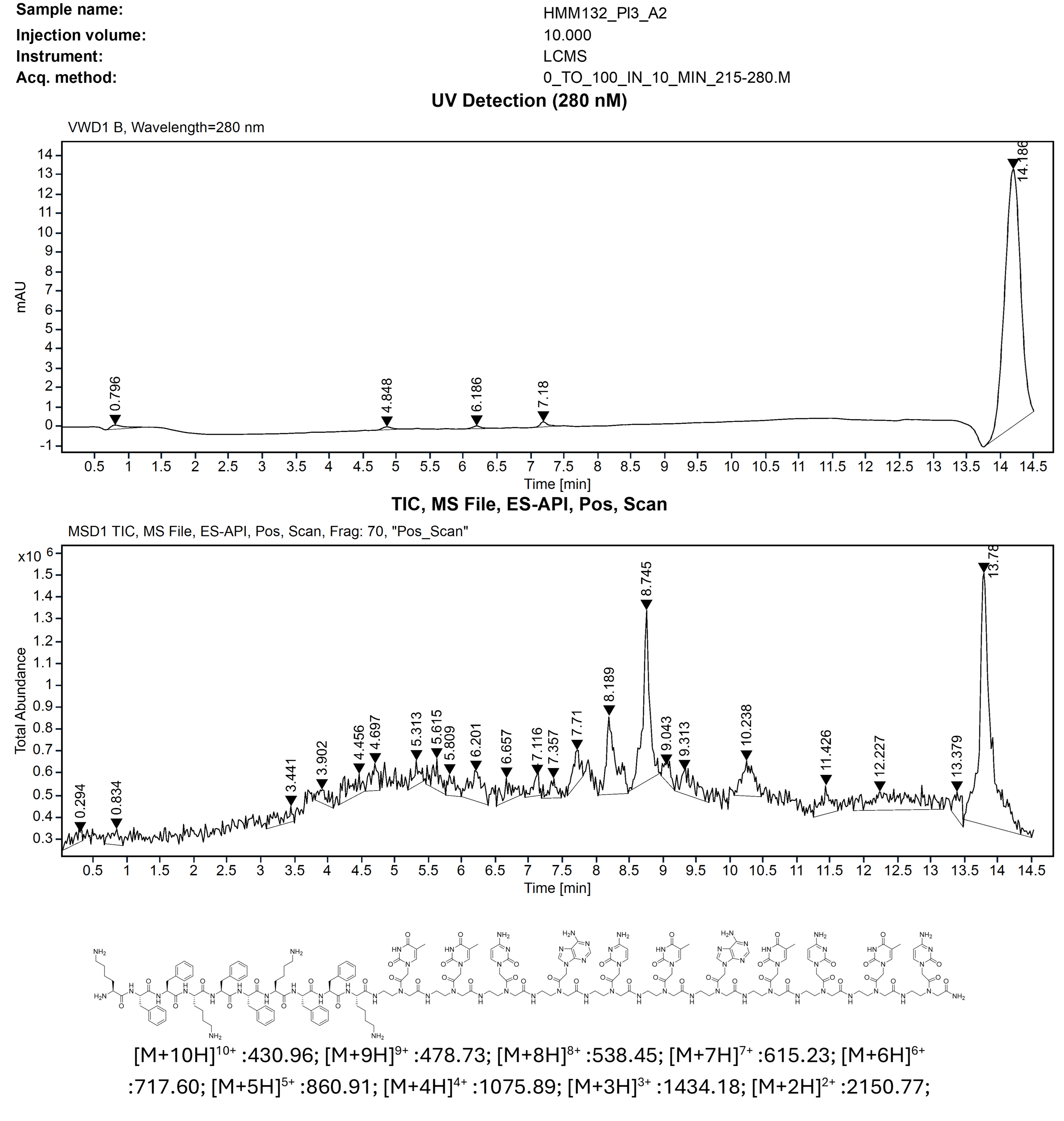

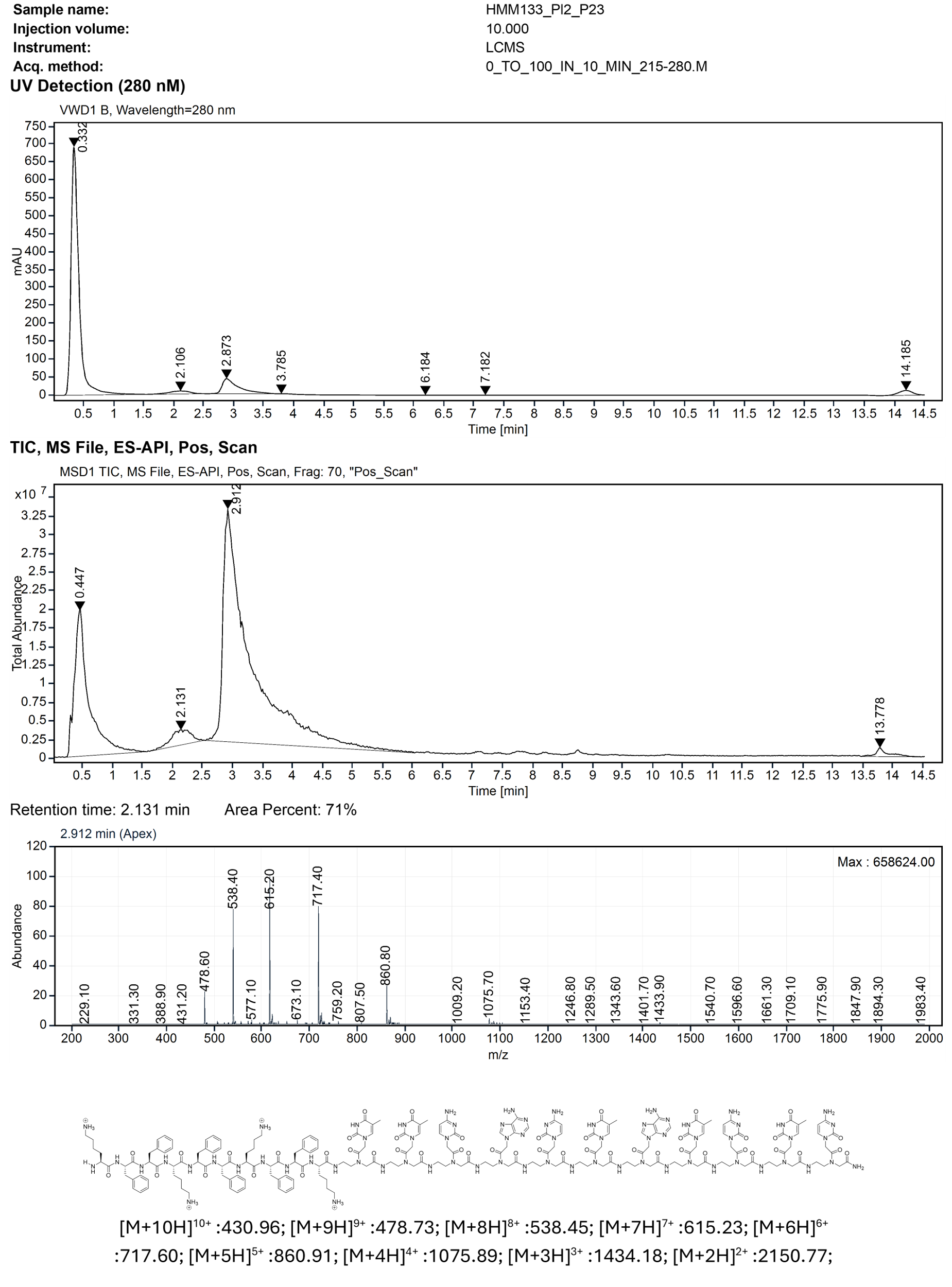

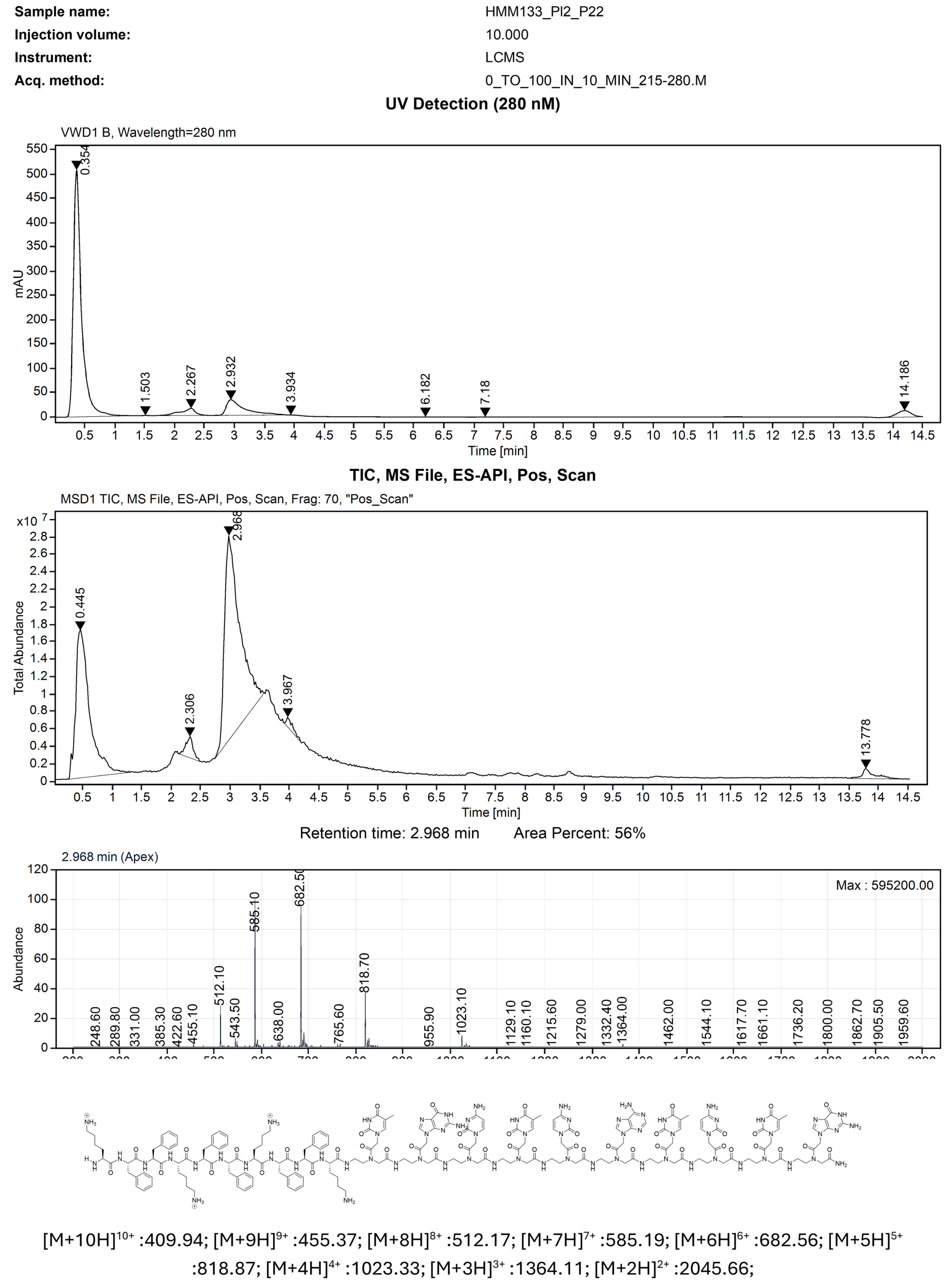

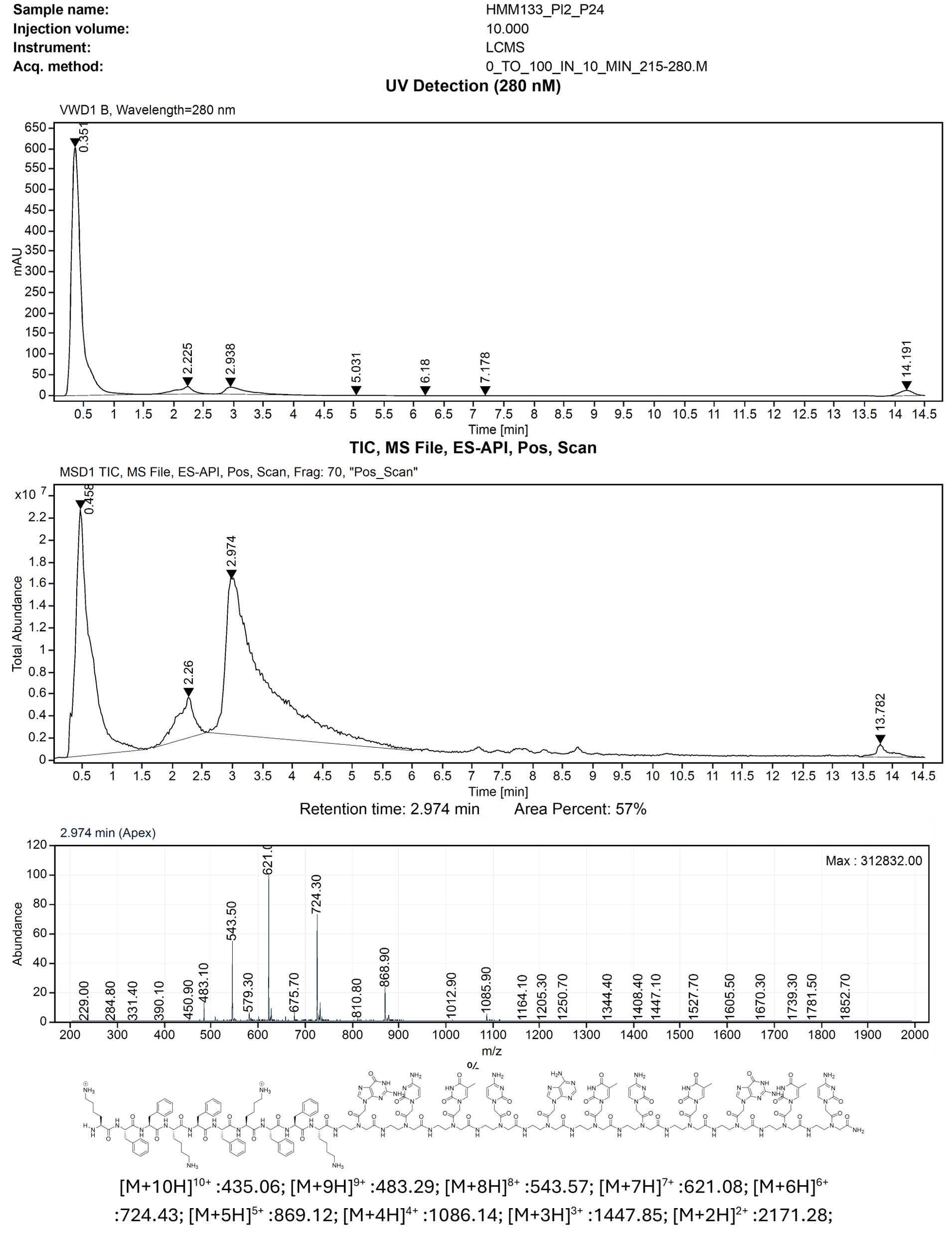

Supplement: Supplementary file 1 — Supporting Information [file ADVS-12-2504284-s001.docx]
